# Photocatalytic Hydrogen Production from Cellobiose Using TiO_2_@Pt(0.5) Immobilized Photocatalyst

**DOI:** 10.1021/acsomega.6c01812

**Published:** 2026-08-09

**Authors:** Bruno C. B. Salgado, Mayara M. R. Oliveira, Sydney F Santos, Jeremy W. J. Hamilton, Kathryn Ralphs, Peter K. J. Robertson

**Affiliations:** † Federal Institute of Ceara., Av. Parque Central, 1315, Maracanau, Ceara 61939-140, Brazil; ‡ Federal University of ABC., Av. dos Estados, 5001, Bangu, Santo André, São Paulo 09.280-560, Brazil; § School of Engineering, Ulster University, 2-24 York Street, Belfast BT15 1AP, United Kingdom; ∥ School of Chemistry and Chemical Engineering, Queen’s University Belfast, Stranmillis Road, Belfast BT9 5AG, United Kingdom

## Abstract

Photocatalytic reforming of biomass-derived carbohydrates is a potentially promising route for sustainable hydrogen production under mild conditions. In this work, we investigated the photocatalytic reforming of cellobiose using TiO_2_@Pt­(0.5) immobilized on a glass plate as a catalytic film prepared by a spray coating method. The immobilized catalyst exhibited a H_2_ production rate of approximately 14 μmol cm^–2^ h^–1^ at 40 °C, achieving performance comparable to slurry-based systems while eliminating the need for postreaction separation of the photocatalyst. Hydrogen evolution yields were primarily governed by photon energy, catalyst loading, and substrate concentration, whereas the cumulative hydrogen yield remained similar within the investigated temperature range (40–80 °C), despite differences in the initial reaction profile and catalyst stability observed at 80 °C. The catalyst demonstrated a stable performance under the tested experimental conditions, maintaining constant activity over 10 consecutive reuse cycles with no detectable loss of immobilized mass. Liquid-phase analysis revealed that the detected products were consistent with oxidative cleavage reactions during cellobiose photoreforming, leading to formic acid and formaldehyde as the main detected products. Overall, the results indicate that the immobilized TiO_2_@Pt­(0.5) photocatalyst demonstrates potential as an effective proof-of-concept material for biomass photoreforming in an immobilized configuration and warrants further optimization and mechanistic investigation.

## Graphical abstract

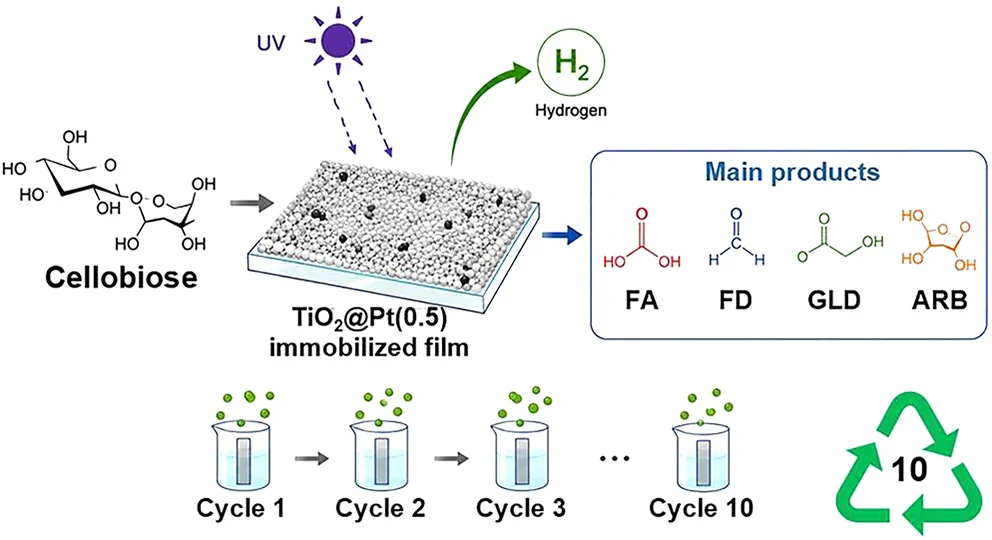

## 1 Introduction

The escalating challenges posed by climate change have highlighted the need for a significant reduction in our fossil fuel dependence, whose extraction and combustion generate substantial greenhouse gas emissions.^1^ In this context, hydrogen (H_2_) has emerged as a very promising energy vector, offering high energy density, zero carbon emissions at the point of use, and broad applicability across industrial and energy sectors.^2,3^ Despite these advantages, global H_2_ production remains predominantly reliant on fossil fuel-based routes, such as steam methane reforming, which are also responsible for significant CO_2_ emissions.^4^ For H_2_ to effectively contribute to the decarbonization of the global economy, the production of green hydrogen must be substantially scaled up.

In recent years, biomass valorization has attracted considerable attention as a promising strategy for the sustainable production of fuels and chemicals.^5,6^ Compared to traditional thermal and biological treatment routes, photocatalysis offers distinct advantages, including operation under mild conditions, reduced energy demand, and the potential for direct utilization of solar energy.^7,8^ In particular, photocatalytic reforming of biomass-derived substrates has emerged as an attractive route for H_2_ production. In this process, organic compounds act as electron donors, consuming photogenerated holes on the photocatalyst, thereby facilitating proton reduction by the photogenerated electrons, resulting in H_2_ evolution under more thermodynamically favorable conditions compared to conventional water splitting.^3,9^

Within the context of organic substrates, an extensive range of different classes of biomass-derived molecules have been studied, including alcohols, carboxylic acids, and saccharides.^10–15^ Recent advances have demonstrated the possibility of photoreforming more complex carbohydrates, such as cellulose, opening new perspectives for the use of lignocellulosic biomass in photocatalytic processes.^16–18^ Cellulose, the most abundant component of lignocellulosic biomass,^2^ is a linear polymer of d-glucose units connected by β-1,4-glycosidic linkages, whose organization into extensive hydrogen bonding networks leads to the formation of highly crystalline and resistant microfibrils.^19^ This high structural stability poses significant challenges for the development of photocatalysts effective at breaking down this structure and for controlling the reaction system, especially when the goal is to convert biomass directly into high-value fuels and chemicals.^18,20^

Although glucose is widely used as a model molecule to investigate cellulose photoreforming,^21,22^ this simple carbohydrate does not allow for a satisfactory elucidation of the mechanisms underlying the photocatalytic cleavage of β-1,4 linkages in the lignocellulosic polymer.^18^ In this context, cellobiose (CLB), a disaccharide consisting of two glucose units linked by the same β-1,4 linkage that constitutes the cellulose chain, is consequently one of the most suitable molecular models for this type of study.^5,23,24^ A detailed understanding of CLB reactivity provides the critical link between studies conducted with simple molecules, such as glucose, and the direct photoreforming of cellulose, representing a fundamental step toward the development of sustainable technologies for converting lignocellulosic biomass into hydrogen.

Most photocatalytic studies to date have been conducted in slurry-type reactors, where the catalyst is dispersed in the reaction medium. While this configuration minimizes mass transfer limitations, it necessitates an additional catalyst separation step, which is time-consuming and costly, particularly when noble metals such as platinum are employed as cocatalysts.^25^ Immobilization of the photocatalyst onto a solid support, such as glass, carbon, or quartz, offers a practical alternative, eliminating the need for postreaction separation and facilitating catalyst recovery and reuse in subsequent cycles.^26–28^ Despite these advantages, studies on the photoreforming of biomass-derived substrates using immobilized catalysts remain scarce. While numerous examples exist for slurry-type photoreforming of biomass over noble metal–supported catalysts,^29–32^ often focusing on catalyst development rather than technological progression, significantly fewer examples report the use of immobilized catalysts, and studies specifically targeting CLB are even rarer.

To date, only one study has reported the photocatalytic reforming of CLB using an immobilized catalyst. Machado et al.^33^ investigated H_2_ production from various saccharides using Pt/g-C_3_N_4_ immobilized on a three-dimensional (3D) support. This investigation demonstrated that CLB was the most promising substrate among those tested, achieving 124 μmol of H_2_ after 180 min reaction time. A previous study by the same group^34^ explored TiO_2_ and CNT-TiO_2_ materials doped with noble metals for the photoreforming of saccharides, including CLB, but without catalyst immobilization. The normalized H_2_ production rate achieved by Machado et al.^33^ was 0.45 μmol cm^–2^ h^–1^. While this work demonstrated the feasibility of immobilized systems for CLB photoreforming, the catalytic activity remained relatively low, indicating considerable room for improvement, and the mechanistic aspects of CLB conversion were also not explored in detail.

In this work, we investigated the photocatalytic reforming of CLB using a TiO_2_@Pt­(0.5) catalyst immobilized on glass plates via a spray coating method. The performance of this immobilized system was evaluated in terms of H_2_ production rate, photocatalyst stability over multiple reuse cycles, and the distribution of liquid-phase products. The results have been compared with a slurry configuration using the same catalyst mass, and with the previously reported immobilized system, to assess the relative performance of the proposed approach. Additionally, the main reaction byproducts have been identified to provide insight into the conversion pathway of CLB under the experimental conditions that were employed.

## 2 Experimental Section

### 2.1 Chemical and Materials

Titanium dioxide (TiO_2_ P25) was supplied by Degussa. Cellobiose (CLB, 98%), glucose (GLU, 99%), fructose (FRT, 99%), glyceraldehyde (GLD, 90%), formaldehyde (FD, 37% aqueous solution), formic acid (FA, 95%), methanol (MeOH, 99.8%), propanol (PropOH, 99.8%), and hexachloroplatinic acid (H_2_PtCl_6_, 99.9%) were obtained from Sigma-Aldrich. Arabinose (ARB, 98%) was acquired from TCI, and a Nafion solution (5%, D520) was supplied by Ion Power.

### 2.2 Synthesis and Immobilization of the Catalyst

The Pt-supported TiO_2_ catalyst was synthesized via photodeposition using hexachloroplatinic acid (H_2_PtCl_6_) as the precursor. A known mass of TiO_2_ P25 was dispersed in an aqueous methanol solution (10% v/v) under magnetic stirring and sonication to ensure homogenization of the suspension. Subsequently, a stoichiometric amount of H_2_PtCl_6_ was added to achieve a final Pt concentration of 0.5% relative to the TiO_2_ mass. The mixture was kept under constant stirring and a nitrogen flow for 2 h, after which it was irradiated with an LED light source (λ = 370 nm) for 3 h. The resulting solid was recovered by centrifugation, washed with deionized water, and dried in an oven at 75 °C for 12 h. The material was then ground and designated as TiO_2_@Pt­(0.5).

For catalyst immobilization, glass slides (2.5 × 7.0 cm^2^) were used after prior cleaning in consecutive ultrasonic baths of water, ethanol, and acetone (30 min each). An immobilization suspension was prepared by dispersing the TiO_2_@Pt­(0.5) catalyst in propanol, using a Nafion solution (5%) as a binding agent, in a volumetric ratio of 1.0:0.5:10.0 (catalyst: Nafion: propanol). Immobilization was carried out via spray coating of the suspension onto the glass slides, which were placed on a heated surface at 75 °C. The deposition area was confined to 6.25 cm^2^. The immobilized catalyst mass was controlled by the number of spray cycles and determined by direct weighing of the slides before and after the procedure. Finally, the catalytic films were dried at 75 °C for 12 h before use.

### 2.3 Characterization

X-ray Diffraction (XRD) analyses were performed on a PANanalytical XPert Pro MPD diffractometer in an angular range of 10–90° (2θ), using a Co Kα radiation source (40 kV and 45 mA). Textural characterization of the synthesized materials was performed through N_2_ adsorption/desorption experiments at −196 °C on Autosorb iQ3 equipment (Quantachrome Instruments). The samples were previously degassed under reduced pressure at 200 °C for 2 h. The BET (Brunauer–Emmet–Teller) and BJH (Barret–Joyner–Halenda) models were adopted for data interpretation. The diffuse reflectance spectrum of the catalysts was obtained in a Thermo Evolution 300 system with spectral scanning from 220 to 800 nm. The band gap energies were determined using the Kubelka–Munk method. High-resolution transmission electron microscopy (HR-TEM), scanning-transmission electron microscopy (STEM) with a high-angle annular dark field (HAADF) detector and Energy Dispersive X-ray spectroscopy (EDX) maps were acquired in a Thermo Scientific microscope, model Talos F200X G2 Scanning Transmission Electron Microscope ((S)­TEM) operating at 200 kV and equipped with an EDX spectrometer (4 detectors) model Super-X EDS. X-ray photoelectron spectroscopy (XPS) analysis was carried out using an ESCALAB XI+ spectrometer (ThermoFisher) equipped with an Al Kα X-ray source (1486 eV) and a spot size of 900 μm, using an operating current of 10 mA and a voltage of 15 kV. Samples were charge neutralized with a flood gun, resulting in a chamber pressure of 10^–8^ bar throughout the measurement process. High-resolution region scans were performed with a resolution of 0.1 eV and a pass energy of 20 eV. The concentration of platinum present in the catalyst was quantified by ICP-OES (Agilent). Photoluminescence (PL) spectra were collected at an excitation wavelength of 320 nm on a fluorescence spectrophotometer (Cary Eclipse) with spectral resolution and slit width set at 1 and 5 nm, respectively. The thickness of the immobilized catalyst film was measured from the cross-section of the catalytic plate. The cleaved plates were coated with a thin layer of gold by sputtering for 45 s in an Emitech k500x instrument to add a conduction path. Scanning electron microscopy (SEM) imaging and film thickness measurement were performed on a Jeol JSM-6010 plus. Samples were mounted vertically using conductive carbon tape on an aluminum stub, wherein tilt ranges of 5° to −1° were used to align the sample for film thickness measurement.

### 2.4 Photocatalytic Reactions

Photocatalytic tests were conducted in a borosilicate glass reactor fitted with a three-port GL45 lid. An aqueous CLB solution (25 mL) was added to the reactor, into which a catalyst-coated plate was vertically immersed. To ensure anoxic conditions, the reaction medium was purged with a nitrogen (N_2_) flow through two of the lid ports for 30 min before irradiation. The third port was fitted with a silicone septum to allow for periodic sampling of the gas phase. An additional sampling port, located on the lower lateral wall of the reactor, was used for liquid phase sampling without interrupting irradiation. Following the purge, the system was hermetically sealed, and the reaction was initiated by irradiation with a 370 nm LED lamp, providing an irradiance of 5.84 mW cm^–2^ (UVP UVX Radiometer). Gas samples were analyzed by GC-TCD using a CP-PoraPLOT U column, while the liquid phase was monitored by HPLC-RI with a Rezex ROA column.

## 3 Results and Discussion

### 3.1 Photocatalyst Characterization

Figure 1 shows HR-TEM (Figure 1.a,b) and HAADF-STEM (Figure 1.c–e) images of the TiO_2_@Pt­(0.5) catalyst. The TiO_2_ particles have an average size of approximately 20 nm (Figure 1.a). The sample exhibited high crystallinity, as observed at higher magnification (Figure 1.b).

**Figure 1:**
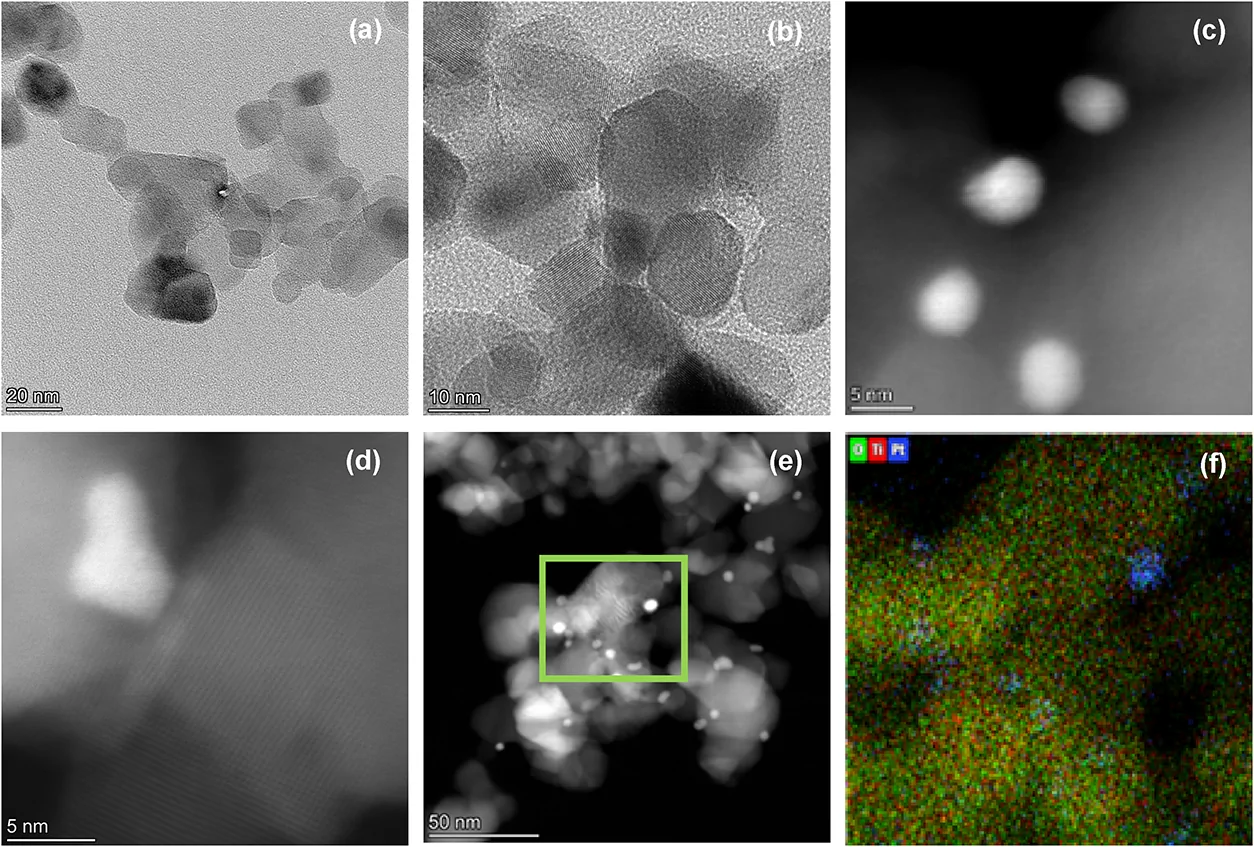
HR-TEM (a,b), HAADF-STEM (c,d,e) images and EDX mapping (f) of the TiO_2_@Pt­(0.5).

The HAADF-STEM images (Figure 1.c,d) make it possible to observe the atomic number contrast (Z contrast) due to different types of inelastic scattering between Ti and Pt, easily detected in the high-angle annular dark field (HAADF). The Pt nanoparticles (NPs) (which have a brighter contrast due to higher Z) with approximately 5 nm are observed to be dispersed onto the TiO_2_ matrix (Figure 1.c). The detailed morphology of one of these NPs is shown in Figure 1.d in which it is observed that the particles are not fully rounded. A low magnification HAADF–STEM image (Figure 1.e) of the microstructure shows the Pt nanoparticles distribution. The green square mark delimits the area analyzed by Energy-dispersive X-ray spectroscopy (EDX). The EDX mapping (Figure 1.f) corroborated the HAADF analysis, i.e., the compositional map clearly indicates the Pt concentration in the small round nanoparticles (blue pixels) while the larger particles are composed of Ti and O (red and green pixels, respectively). Figure S1 shows the individual elemental mapping. In support of these findings, ICP-OES analysis revealed a Pt concentration in the catalyst of 0.48% (w/w), highlighting the high efficiency of the photodeposition method employed in this study. Taken together, the HR-TEM, HAADF-STEM, EDX and ICP-OES results suggest that Pt was successfully deposited as highly dispersed nanoparticles preferentially located at the external surface of TiO_2_.

The textural properties of the catalysts were evaluated through N_2_ adsorption/desorption isotherms using the BET and BJH methods (Figure S2). The isotherm profiles are characteristic of type IV, typical of mesoporous materials, with a hysteresis loop associated with type H3, commonly observed in materials composed of aggregates of nearly spherical or lamellar particles. The data in Table S1 show that the surface area remained practically unchanged after Pt deposition. In contrast, a marked increase in both pore diameter and pore volume was observed. This result may be associated with the presence of Pt nanoparticles, which could induce modifications in the packing arrangement of TiO_2_ particles or contribute to the enlargement of interparticle spaces, as suggested by the HAADF-STEM images (Figure 1), where Pt nanoparticles are homogeneously dispersed without significant agglomeration. It should, however, be noted that the interpretation of textural changes in composite systems such as TiO_2_@Pt­(0.5) is not straightforward, and other factors, including possible variations in particle aggregation during catalyst preparation, may also influence the observed pore structure. The textural results are broadly consistent with the microscopy observations. While the specific surface area remained essentially unchanged after Pt deposition, the increase in pore diameter and pore volume may reflect subtle modifications in particle packing associated with the presence of dispersed Pt nanoparticles. Nevertheless, the available data does not allow the origin of these textural changes to be unambiguously established, and contributions from variations in particle aggregation cannot be excluded.

The presence of Pt as nanoparticles did not induce changes in the crystalline structure compared to TiO_2_ P25, as shown in Figure 2.a. The materials exhibit anatase and rutile crystal phases in a proportion of 80:20, respectively. Despite the maintenance of the crystalline phases, a slight leftward shift is observed in the diffraction pattern of the Pt-containing material, particularly for the most intense peaks associated with the anatase and rutile phases at 2θ of 29.7° (101) and 32.1° (110), respectively, as highlighted in Figure 2.b. Considering the low Pt loading (0.5%) and the small average nanoparticle size (∼5 nm), no formation of new crystalline phases or reflections directly attributed to metallic Pt is observed.

**Figure 2:**
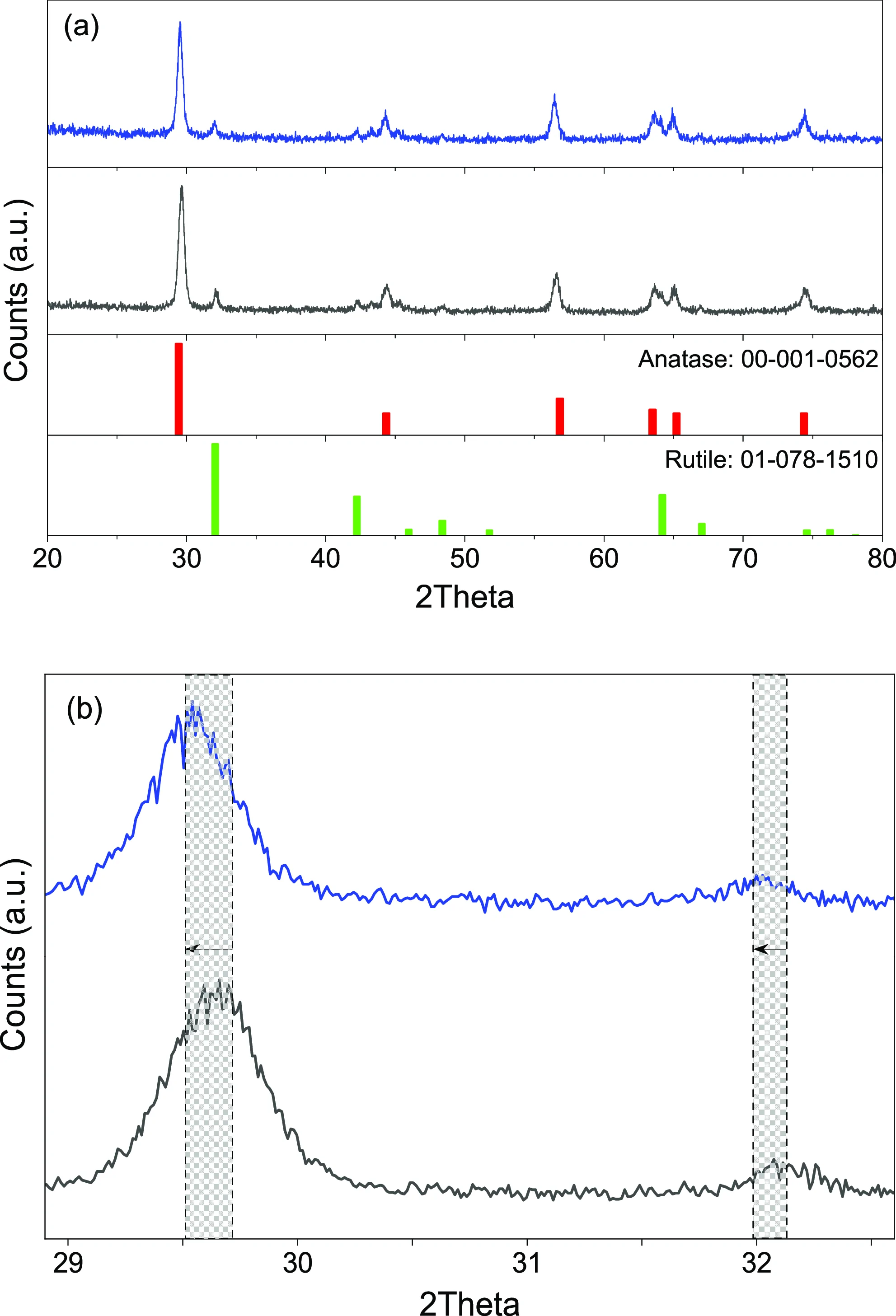
(a) XRD profiles of TiO_2_ P25 (black) and TiO_2_@Pt­(0.5) (blue), and (b) peak shift due to platinum coating.

Similar peak shifts have been reported in the literature for Pt–TiO_2_ systems with low metal content and high dispersion.^35^ One possible interpretation is that local lattice distortions near the Pt–TiO_2_ interface, arising from electronic interactions or mechanical strain, may lead to slight variations in interplanar distances that manifest as peak shifts.^36^ When considered together with the TEM observations, these results suggest that Pt deposition occurs predominantly at surface or interface regions rather than through incorporation into the TiO_2_ lattice. The absence of additional crystalline phases and the preservation of the anatase/rutile ratio indicate that the photodeposition procedure largely preserves the crystal structure of TiO_2_ while introducing modifications at the metal–semiconductor interface.

X-ray photoelectron spectroscopy was used to determine the surface composition and oxidation states of elements present in TiO_2_@Pt­(0.5) photocatalyst. High resolution XPS spectra in the Ti 2p, O 1s and Pt 4f binding energy regions of the investigated TiO_2_@Pt­(0.5) are reported in Figure 3. The O 1s region (Figure 3.a) exhibits a double peak; deconvolution indicates the presence of lattice oxygen (530.0 eV) and surface oxygen (531.8 eV), in good agreement with the literature.^37–39^ The contribution of binding energy centered at 531.8 eV may be associated with surface hydroxyl groups, adsorbed oxygen species, and/or oxygen-deficient environments related to surface defects or oxygen vacancies.

**Figure 3:**
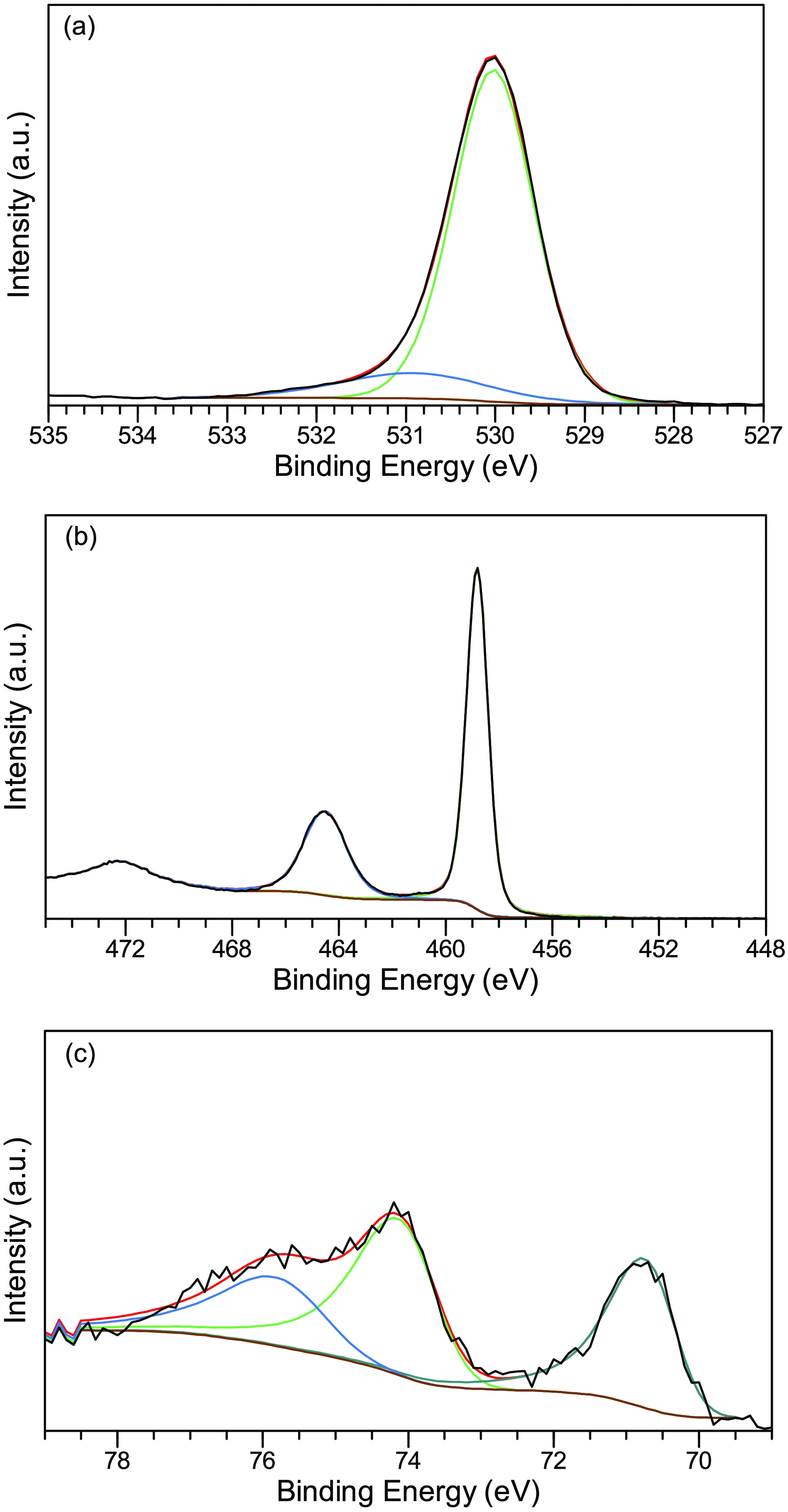
High resolution XPS spectra for (a) O 1s, (b) Ti 2p and (c) Pt 4f for the TiO_2_@Pt­(0.5).

Two peaks were detected in the Ti 2p region (Figure 3.b), with energies centered at 459.3 and 465.0 eV, assigned to Ti 2p_3/2_ and Ti 2p_1/2_, respectively. The energy separation between these peaks is 5.7 eV, characteristic of the Ti^4+^ chemical state in TiO_2_.^40,41^ It is important to emphasize that no significant changes were observed in the binding energies of Ti 2p nor substantial changes in the peak shape after Pt photodeposition, as evidenced by the comparison presented in Figure S3. This behavior suggests that Pt deposition did not significantly alter the electronic structure of the TiO_2_ lattice under light-free conditions and corroborates the interpretation that Pt is deposited predominantly on the catalyst surface, rather than being incorporated into the TiO_2_ crystal structure. This interpretation is also consistent with the XRD results, which showed preservation of the crystalline phases after Pt deposition. Together, the XRD and Ti 2p XPS analyses indicate that the bulk TiO_2_ framework remains largely unchanged, while the effects of Pt deposition are primarily associated with the catalyst surface.^42^

The Pt 4f region spectrum (Figure 3.c) was analyzed and fitted with a doublet, with a separation of 3.33 eV between the 4f_7/2_ and 4f_5/2_ peaks, located at 71.1 and 74.4 eV, respectively, characteristic of metallic platinum on the surface.^43^ Additionally, a peak at 75.7 eV is observed, which can be attributed to the presence of Pt^2+^.^44^ Analysis of the obtained areas indicates that platinum is distributed between the Pt^0^ and Pt^2+^ states in a ratio of 71:29, respectively. This result demonstrates that the proposed photodeposition method was highly effective in reducing Pt^4+^ ions present in the platinum precursor salt, leading to the formation of a substantial fraction of metallic Pt^0^ species on the TiO_2_ surface. The high proportion of Pt^0^ is particularly advantageous for the photocatalytic production of hydrogen, since metallic platinum acts as an efficient electron accumulation and proton reduction site, promoting the conversion of H^+^ to H_2_.

Quantitative XPS analysis (Table S2) revealed a Pt surface atomic concentration of 0.23%. After converting the atomic percentage obtained by XPS into its corresponding mass-based representation for comparison with ICP results, the Pt concentration determined by XPS was found to be higher than that obtained by ICP analysis (1.8% and 0.48% w/w, respectively). This behavior is consistent with the distinct analytical depths of the two techniques, since ICP measures the overall bulk composition of the catalyst, whereas XPS is inherently surface-sensitive and probes only the outermost atomic layers of the material. Therefore, the comparatively higher Pt concentration detected by XPS indicates that Pt species are preferentially concentrated at the TiO_2_ surface rather than distributed throughout the bulk. This interpretation is consistent with the TEM and HAADF-STEM images, which revealed Pt nanoparticles predominantly located at the external surface of TiO_2_ particles.

The optical behavior of the materials was investigated by diffuse reflectance spectroscopy (DRS), and the corresponding spectra are presented in Figure 4. The optical band gap energies were experimentally determined from the reflectance data using the Kubelka–Munk function. Given the indirect nature of the electronic transition in TiO_2_, the band gap values were obtained from the linear extrapolation of the plots of [F­(R)·hν]^1/2^ as a function of photon energy (hν). The pristine TiO_2_ exhibited a band gap energy of 3.32 eV, while TiO_2_@Pt­(0.5) showed an apparent reduction to 3.05 eV. This change in the optical response may reflect interfacial effects associated with Pt deposition, influencing the effective optical transitions of the composite material. In particular, the interaction between the metal and the semiconductor leads to changes in the effective optical transitions of the composite system, consistent with metal–semiconductor junction formation. When considered together with the structural and surface characterization results, these optical changes appear to be associated primarily with the Pt–TiO_2_ interface. Since both XRD and Ti 2p XPS suggest that the crystalline framework and chemical state of TiO_2_ remain largely unchanged after Pt deposition, the observed modifications in the optical response are more likely related to interfacial interactions between Pt and TiO_2_ than to substantial structural changes in the semiconductor.

**Figure 4:**
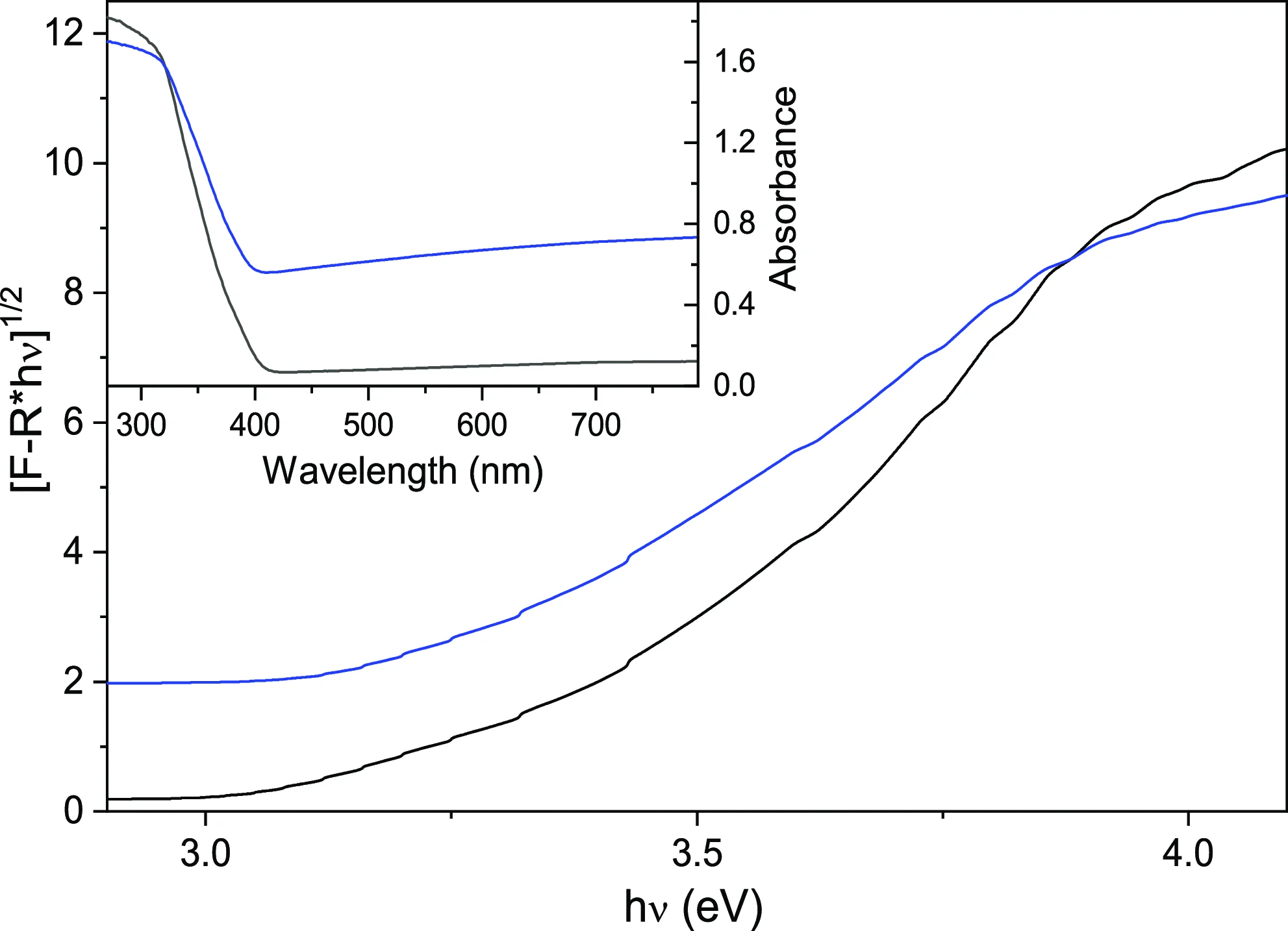
UV–vis diffuse reflectance spectrum of TiO_2_ P25 (black) and TiO_2_@Pt­(0.5) (blue).

To provide a framework for discussing charge transfer processes, the positions of the valence band (VB) and conduction band (CB) edges were estimated using the Mulliken electronegativity approach in combination with the experimentally determined band gap energies.^39^

These estimates, summarized in Table 1, should be regarded as approximate theoretical values derived from bulk electronegativity parameters and experimentally determined band gap energies. They are intended solely to provide a qualitative reference for discussing possible charge-transfer pathways and do not constitute direct measurements of the electronic structure at the TiO_2_@Pt­(0.5) interface. The deposition of Pt gives rise to the formation of a Schottky junction at the Pt–TiO_2_ interface due to the mismatch between the work function of metallic Pt (∼5.65 eV) and that of TiO_2_ (∼4.2 eV for anatase). This junction is expected to generate an internal electric field that may facilitate the transfer of photogenerated electrons from the TiO_2_ conduction band to the Pt nanoparticles, while holes remain in the valence band of the semiconductor, as illustrated in Figure 5. Such interfacial charge separation provides a plausible framework for interpreting the optical behavior of the material.

**Figure 5:**
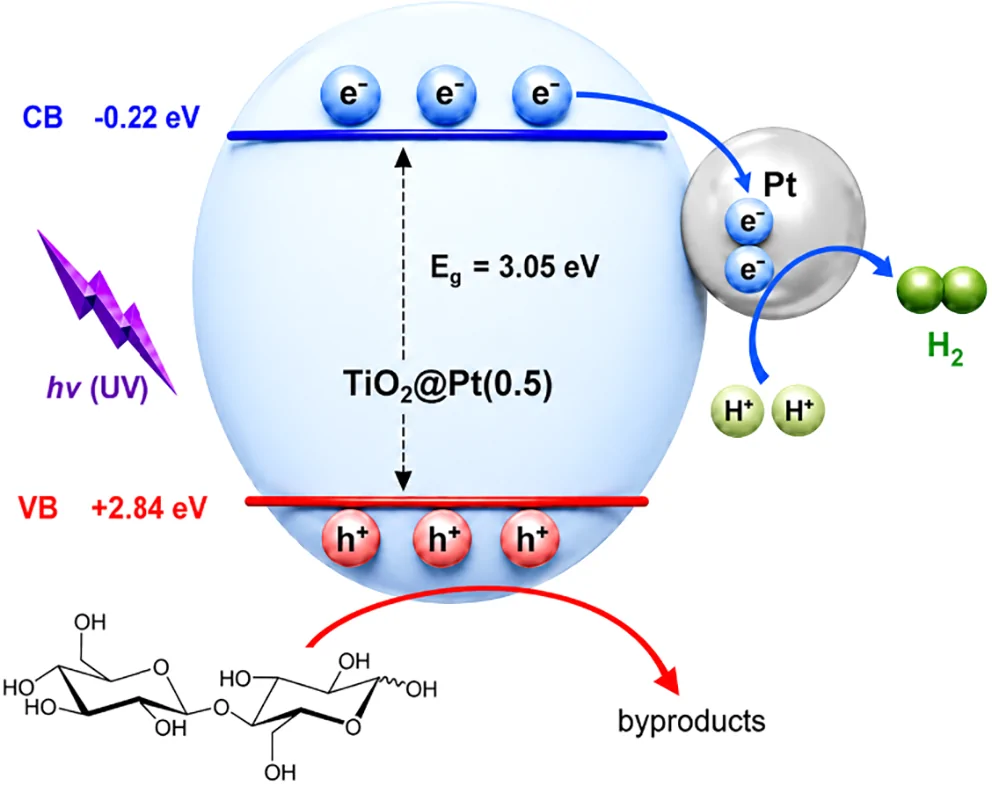
Illustrative scheme of Schottky junction in TiO_2_@Pt­(0.5).

**Table 1:** Energy Levels of the Band-Gap and of the Conduction and Valence Bands of TiO_2_ and TiO_2_@Pt­(0.5)

catalyst	E_g_ (eV)	E_VB_ (eV)	E_CB_ (eV)
TiO_2_ P25	3.32	2.97	–0.35
TiO_2_@Pt(0.5)	3.05	2.84	–0.22

The photoluminescence (PL) spectrum of pure TiO_2_ (P25) and TiO_2_@Pt­(0.5) is shown in Figure S4. The recombination rate of the photogenerated charge carriers is a crucial parameter for defining the photocatalytic activity of a material, with high photocatalytic performance being attributed when such recombination is reduced.^44^ The photoluminescence of a material is directly related to the recombination of the photogenerated electron–hole pair; that is, the intensity of the photoluminescence signal of a photocatalyst is proportional to its recombination rate.^45^

When analyzed together with the DRS, XPS, XRD and electron microscopy results, the PL data complete a coherent description of the TiO_2_@Pt­(0.5) material. The microscopy and XPS analyses suggest that Pt is preferentially located at the catalyst surface, XRD indicates preservation of the TiO_2_ crystalline framework, and DRS reveals modifications in the optical response that may be associated with the metal–semiconductor interface. Within this context, the lower PL intensity observed for TiO_2_@Pt­(0.5) is consistent with changes in charge-carrier dynamics resulting from these interfacial interactions. Although PL measurements do not directly identify charge-transfer pathways, the combined characterization results suggest that Pt deposition influences both the surface chemistry and the electronic behavior of the composite material.

### 3.2 Photocatalytic Activity

Figure S5 shows the wavelength-dependent photocatalytic performance of TiO_2_@Pt­(0.5) for H_2_ production from CLB, revealing a dependence of activity on the energy of the incident photons. The highest hydrogen evolution rate was obtained under ultraviolet irradiation (370 nm), which is consistent with the optical characterization results indicating that the most efficient electronic excitation occurs when the photon energy exceeds the band gap threshold of the TiO_2_@Pt­(0.5) material. Upon irradiation at 400 nm, hydrogen production was still observed, although at a reduced rate.

This response suggests that photons with energies close to the absorption edge of TiO_2_@Pt­(0.5) can initiate electronic excitation, leading to the formation of electron–hole pairs. In this near-threshold regime, the persistence of measurable H_2_ production may be associated with the interfacial electronic interactions discussed in Section 3.1. In particular, the DRS and PL results suggest that Pt deposition influences charge-carrier dynamics at the TiO_2_ surface, which may contribute to maintaining photocatalytic activity even when excitation occurs close to the absorption edge. In contrast, no measurable photocatalytic activity was detected under visible-light irradiation at 440 nm. This observation is consistent with the optical and electronic properties of the material derived from DRS analysis (Figure 4) and summarized in Table 1, supporting the interpretation that photon energy below the absorption edge is insufficient to promote electronic excitation in the catalyst. The band gap energy of TiO_2_@Pt­(0.5) is 3.05 eV, corresponding to an absorption edge of approximately 406 nm. Consequently, photons with energies below this threshold are insufficient to promote excitation in the catalyst, rendering charge generation ineffective despite the presence of Pt.

Catalytic plates with different masses of immobilized catalyst were prepared by spray coating (Figure S6) and evaluated for hydrogen production. The results presented in Figure 6 demonstrate a proportional relationship between the mass of immobilized catalyst and the amount of H_2_ produced, suggesting that increasing catalyst loading increases the amount of photocatalytically accessible material available for light absorption and interfacial reactions.

**Figure 6:**
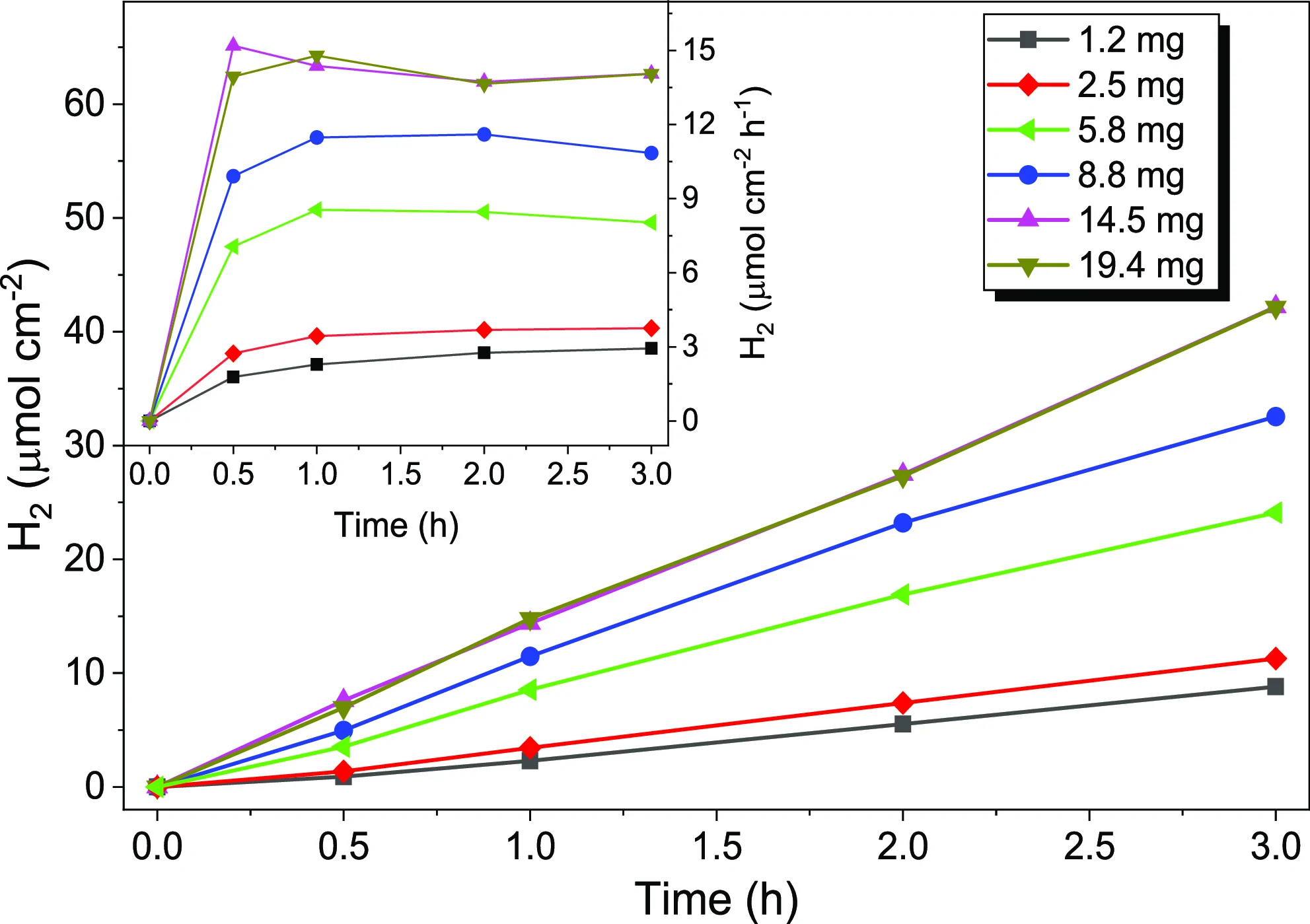
Effect of immobilized catalyst mass on photocatalytic performance for H_2_ production. Catalyst: TiO_2_@Pt­(0.5), [CLB] = 50 mmol L^–1^, lamp 370 nm, *T* = 40 °C.

A threshold was observed at a catalyst mass of approximately 14.5 mg, above which further increases in catalyst loading did not result in additional H_2_ production. One possible explanation is that beyond this loading, the increased film thickness may reduce light penetration to the deeper catalyst layers, potentially limiting the effective utilization of the photocatalyst. Additionally, mass and charge transport within thicker films may become increasingly restrictive, which could affect overall catalytic efficiency.^46^

Additionally, the inset graph in Figure 6 shows the normalized H_2_ production rate as a function of time, demonstrating that, regardless of the catalyst mass employed, the catalytic activity remained constant throughout the reaction time. This behavior indicates high operational stability of the immobilized TiO_2_@Pt­(0.5) system, suggesting no significant deactivation of active sites during the reaction.

Although the spray coating method provides good catalyst distribution on the plate surface, it is evident that the deposition of multiple catalyst layers is necessary to achieve maximum performance, allowing a critical mass of active material to be reached without excessively compromising light transmission or reagent accessibility.^47,48^ Figure S7 shows SEM image of a cross-section of the photocatalytic plate containing 14.5 mg of TiO_2_@Pt­(0.5), revealing that the immobilized film thickness is approximately 20 μm.

Figure 7 shows the effect of the initial CLB concentration on hydrogen production. The amount of H_2_ produced increased with CLB concentration, indicating that substrate availability influences the overall productivity of the photocatalytic process under the investigated conditions.

**Figure 7:**
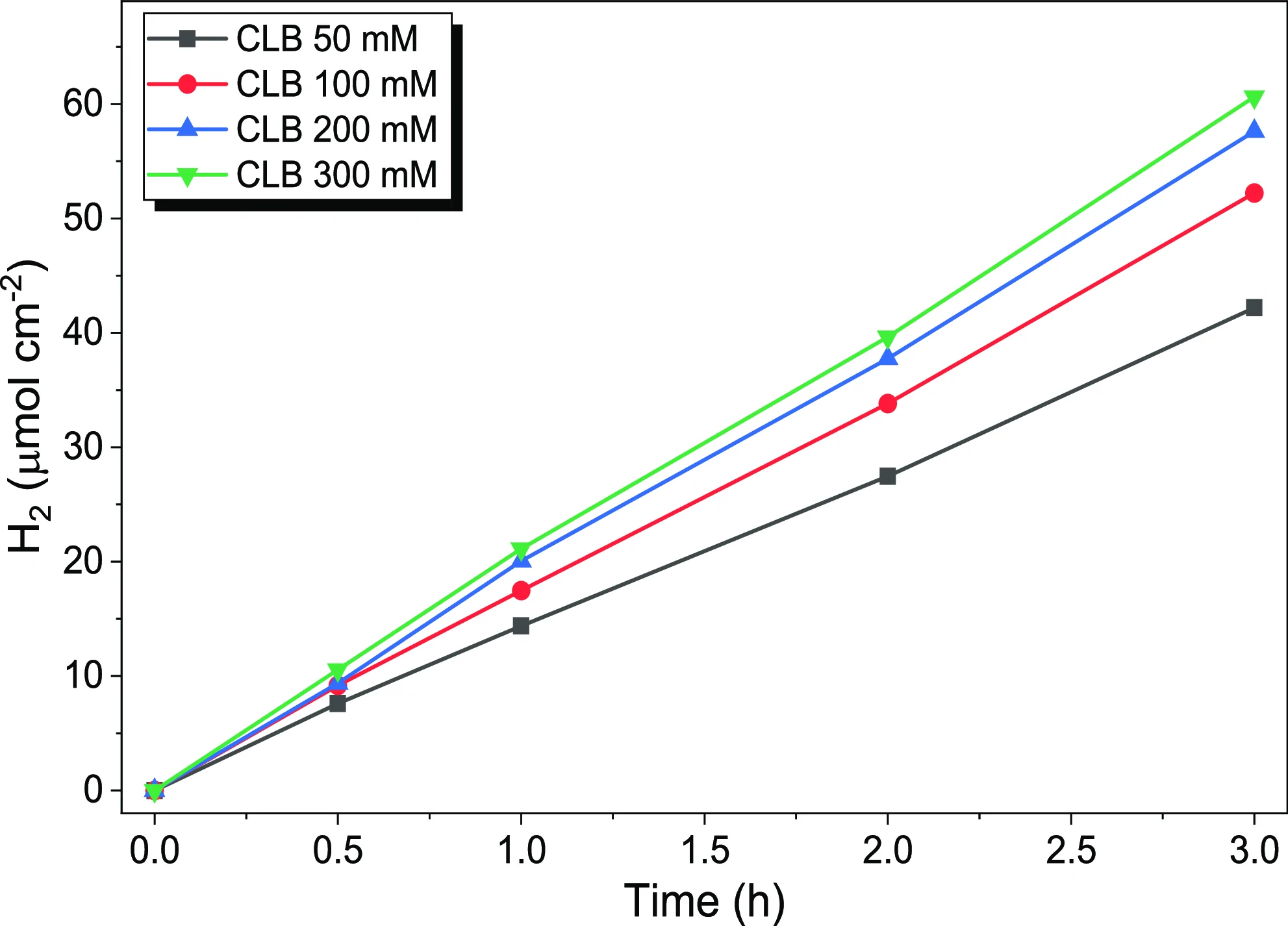
Effect of CLB concentration on the photocatalytic performance for H_2_ production. Catalyst: TiO_2_@Pt­(0.5), immobilized catalyst mass = 14.5 mg, lamp 370 nm, *T* = 40 °C.

This indicates that the immobilized catalyst mass (14.5 mg) had sufficient active sites available to effectively act in the photocatalytic reforming of CLB. This result is consistent with the catalyst-loading experiments, which indicated that 14.5 mg corresponds to a region of efficient catalyst utilization. Within this operating window, increasing substrate concentration resulted in increased hydrogen production, suggesting that the catalyst surface remained capable of processing additional reactant molecules. Increasing the CLB concentration from 200 to 300 mmol L^–1^ did not, however, increase the amount of H_2_ produced to the same extent, suggesting saturation of the catalytic surface. It should be noted that higher concentrations were not evaluated due to the solubility limit of CLB in the aqueous phase.

The H_2_ evolution profile shows linear behavior throughout the reaction time, consistent with the catalytic stability of TiO_2_@Pt­(0.5) previously reported in the evaluation of the immobilized catalyst mass. Machado et al.^33^ reported a study of CLB conversion using platinum-doped carbon nanotubes (Pt/GCNT) immobilized in an area of 88 cm^2^, showing a production of approximately 120 μmol of H_2_ in 3 h of reaction for an initial CLB concentration of 50 mM, corresponding to a normalized rate of 0.45 μmol cm^–2^ h^–1^. In the present case, the reaction rate of H_2_ production found with the TiO_2_@Pt­(0.5) catalyst immobilized in a catalytic area of 6.25 cm^2^ for the same CLB concentration was equivalent to 14.01 μmol cm^–2^ h^–1^. These results indicate that the TiO_2_@Pt­(0.5) immobilized film achieved comparatively high H_2_ production rates under the investigated conditions.

Figure 8 compares the photocatalytic performance of TiO_2_@Pt­(0.5) in slurry and immobilized configurations, using the same catalyst mass (14.5 mg) in a 50 mmol L^–1^ CLB solution. Under the conditions employed, the total H_2_ production after 3 h of irradiation was comparable between the two configurations, with the immobilized system achieving a normalized production rate of approximately 14 μmol cm^–2^ h^–1^. This result demonstrates that catalyst immobilization did not significantly compromise hydrogen production under the experimental conditions investigated.

**Figure 8:**
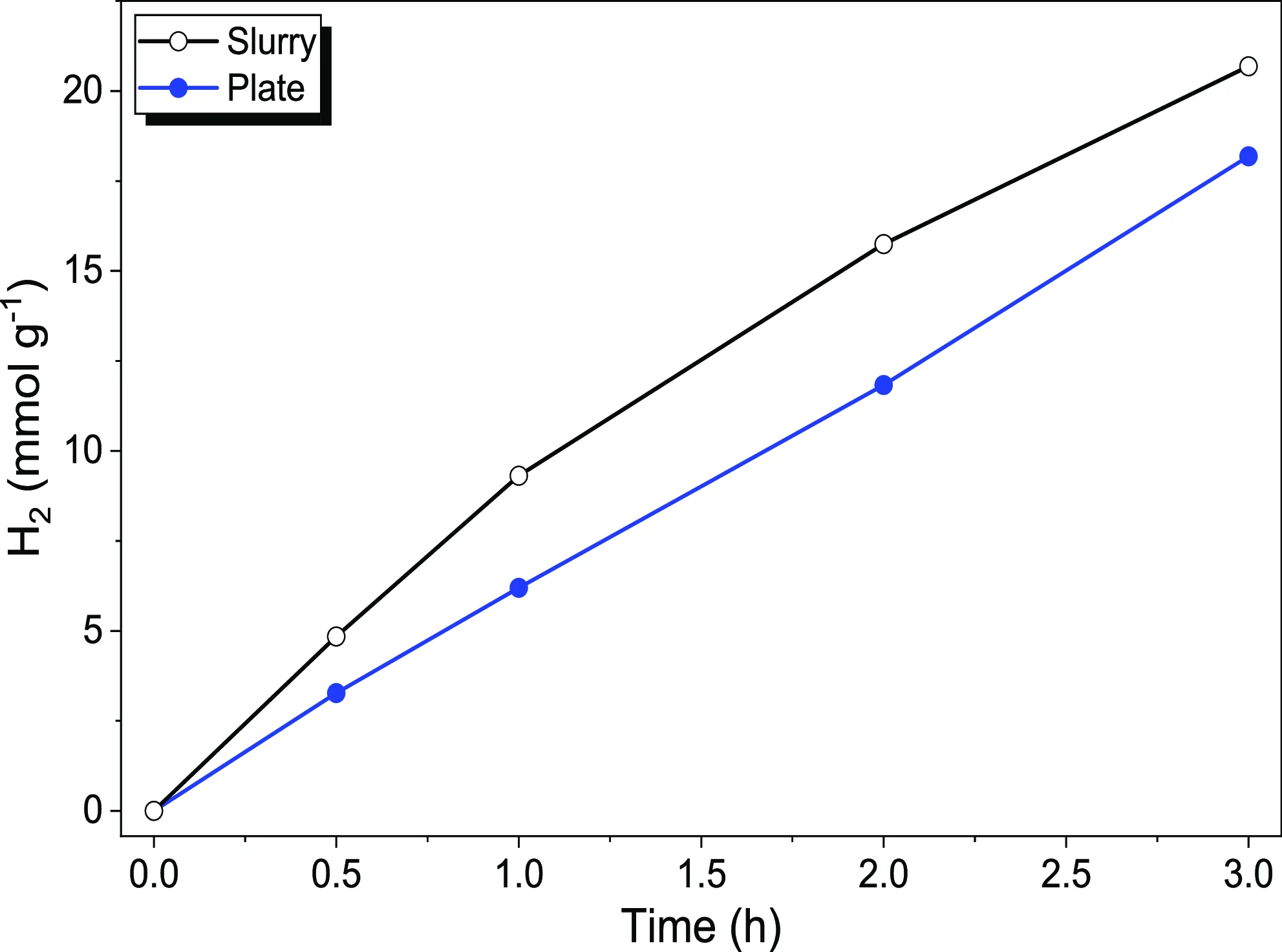
Comparison of H_2_ production performance of TiO_2_@Pt­(0.5) catalyst applied in dispersion (slurry) or immobilized (plate). Catalyst: TiO_2_@Pt­(0.5), mass of the catalyst in suspension and immobilized = 14.5 mg, [CLB] = 50 mmol L^–1^, lamp 370 nm, *T* = 40 °C.

It is important to note, however, that direct comparison between slurry and immobilized systems is not straightforward, as the two configurations differ fundamentally in terms of catalyst distribution, light–catalyst interaction, and mass transport characteristics. In the slurry system, the catalyst particles are dispersed throughout the reaction volume, which generally favors substrate–catalyst contact but can introduce optical attenuation due to light scattering and absorption by suspended particles. In contrast, in the immobilized system, the catalyst is confined to a fixed film, and the incident radiation must penetrate only the film thickness rather than the entire suspension, potentially leading to more efficient photon utilization provided that the film is sufficiently thin to avoid self-shadowing.^49^ In the present study, the immobilized catalyst mass (14.5 mg deposited over 6.25 cm^2^) corresponds to a film thickness that appears to balance catalyst loading with light penetration, as suggested by the threshold behavior observed in Figure 6. While the comparable H_2_ production rates between the two configurations indicate that the immobilized film was able to achieve effective catalyst utilization under the tested conditions, it should be acknowledged that this interpretation assumes that the intrinsic catalytic activity per unit mass is equivalent in both configurations. Factors such as differences in local photon flux, mass transfer limitations, and potential variations in catalyst accessibility within the film could influence the observed performance and warrant further investigation. The comparable H_2_ production rates obtained for the slurry and immobilized systems indicate that both configurations could promote CLB photoreforming effectively under the investigated conditions. Nevertheless, because factors such as local photon distribution, catalyst accessibility, film architecture, and mass-transfer limitations were not independently quantified, the present results should be interpreted as evidence of comparable overall performance rather than as an indication of equivalent intrinsic photocatalytic behavior. Further studies involving optical characterization and reactor analysis would be necessary to establish the relative contributions of light utilization and transport phenomena to the performance of each configuration. Beyond catalytic performance, the immobilized configuration offers practical advantages in terms of operational versatility, as it eliminates the need for postreaction catalyst separation and facilitates catalyst recovery and reuse. Similar results were obtained by Goto et al.^50^ in a study on photocatalytic water splitting using Al-doped SrTiO_3_ as catalyst.

The effect of temperature on the photocatalytic performance of TiO_2_@Pt­(0.5) was investigated, evaluating H_2_ production at 40, 60, and 80 °C, as shown in Figure S8. The results indicate that the temperature variation did not significantly influence the cumulative H_2_ production at the end of the 3 h reaction, with comparable values obtained under all tested conditions. No difference in kinetic profile was observed between 40 and 60 °C. At 80 °C, however, a more favorable reaction kinetics was observed in the initial stages, with a decrease in the reaction rate as the reaction progressed, as shown in the inserted graph in Figure S8. This behavior suggests that increasing temperature may influence the transient reaction kinetics, even though the final hydrogen yield remained comparable after 3 h of irradiation. It is worth noting that at 80 °C, a small loss of immobilized catalyst was observed, suggesting that operation at elevated temperatures may not necessarily provide practical advantages, especially considering that comparable hydrogen yields were obtained at lower temperatures where the mechanical stability of the film was preserved. These results indicate that, within the investigated range, photocatalytic hydrogen production is influenced more strongly by photon-driven processes than by moderate variations in reaction temperature. This interpretation is consistent with the wavelength-dependence experiments, which revealed a pronounced sensitivity of the system to photon energy.

The catalytic stability of immobilized TiO_2_@Pt­(0.5) was evaluated through reuse tests in consecutive photocatalytic H_2_ production cycles, using a plate containing the optimized catalyst mass. Between each cycle, the plate was only washed with deionized water, dried, and weighed to monitor any mass loss of the immobilized catalyst. The results obtained are presented in Figure 9.

**Figure 9:**
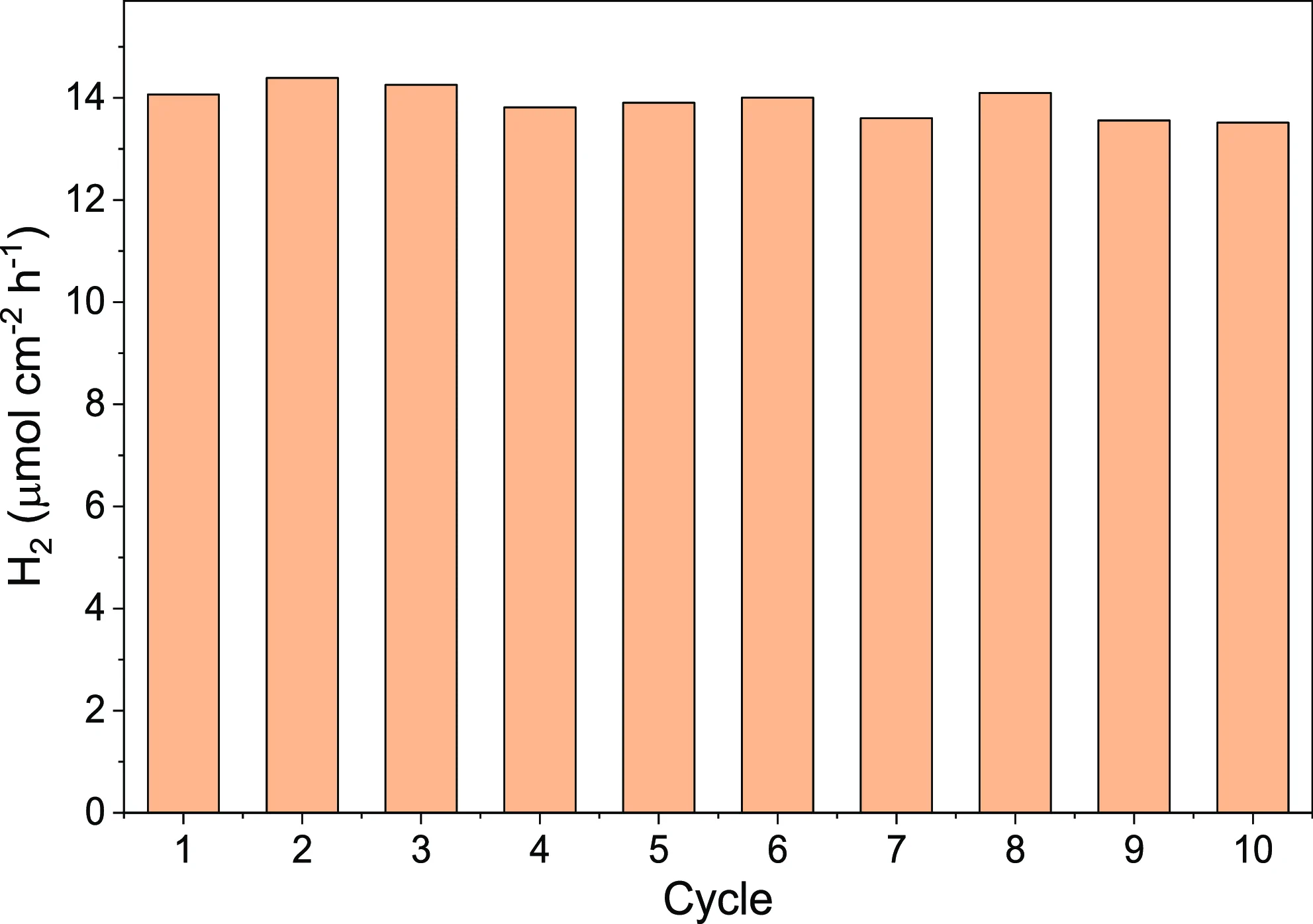
Stability of H_2_ production of catalytic plates in reuse cycles. Catalyst: TiO_2_@Pt­(0.5), [CLB] = 50 mmol L^–1^, immobilized catalyst mass = 14.5 mg, lamp 370 nm, *T* = 40 °C, reaction time = 3 h.

The immobilized TiO_2_@Pt­(0.5) exhibited stable photocatalytic performance throughout the reuse experiments, with no substantial variation in hydrogen production over ten consecutive cycles, with an average production of approximately 14 μmol cm^–2^ h^–1^, without significant variation in activity after 10 consecutive reuses. Additionally, no change in the mass of the immobilized catalyst was detected, indicating that the immobilization method applied proved to be efficient in maintaining the mechanical integrity of the plates. The maintenance of photocatalytic activity throughout the reuse cycles further suggests that no major deactivation phenomena occurred during the investigated period, with no detectable loss of immobilized catalyst mass and no decline in photocatalytic activity over 10 reuse cycles. Although Pt leaching was not directly quantified, the combination of stable photocatalytic performance and the absence of measurable catalyst mass loss is consistent with limited Pt loss under the conditions employed. This behavior is particularly relevant, since the loss of noble metal cocatalysts is frequently cited as one of the main deactivation factors in photocatalytic systems. These results indicate that the immobilized photocatalyst can be reused over multiple consecutive cycles without substantial loss of activity under the investigated conditions. In addition, the immobilized configuration facilitates catalyst recovery and may be advantageous for future studies focused on process optimization and fundamental reactor development.

### 3.3 Byproducts of the Photocatalytic Conversion of Cellobiose

Figure 10 shows the monitoring of CLB conversion products in the aqueous phase. At the end of the 3 h reaction period, the immobilized TiO_2_@Pt­(0.5) led to a conversion of 5.4% of CLB at an initial concentration of 50 mmol L^–1^, corresponding to 2.7 mmol L^–1^ of CLB converted (32.4 mmol L^–1^ of carbon).

**Figure 10:**
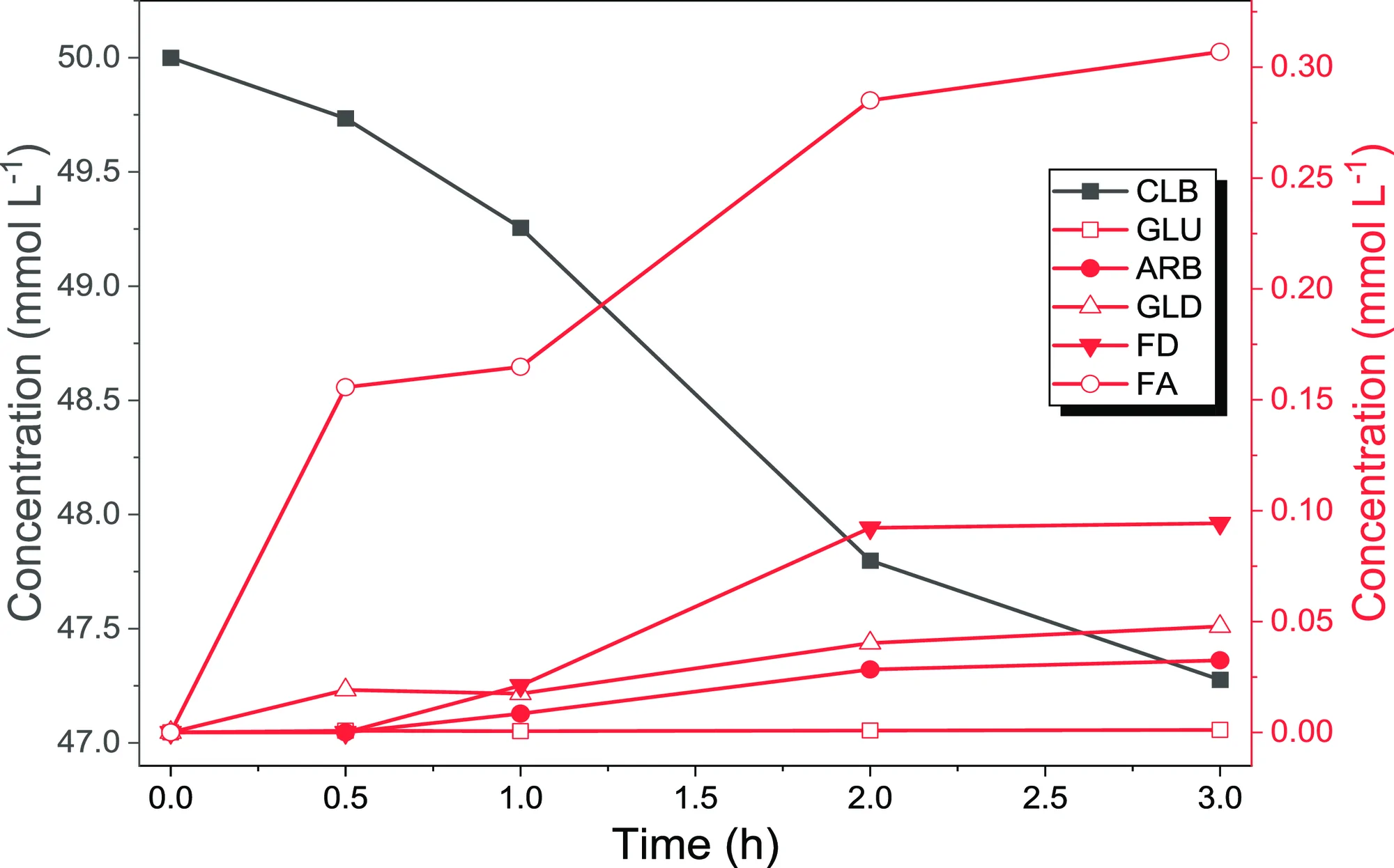
Monitoring of photocatalytic conversion byproducts of CLB in aqueous phase. Catalyst: TiO_2_@Pt­(0.5), [CLB] = 50 mmol L^–1^, immobilized catalyst mass = 14.5 mg, lamp 370 nm, *T* = 40 °C.

Formic acid and formaldehyde were the major products detected in the liquid phase. The predominance of these low molecular weight compounds is consistent with a deep oxidative cleavage pathway, rather than the accumulation of larger glycidic intermediates.^51^ One plausible interpretation is that the cleavage of the β-1,4-glycosidic bond is followed by consecutive oxidation and C–C cleavage reactions. In this context, the initial decomposition of CLB may yield glucose units, which could undergo further oxidation to intermediates such as arabinose and glyceraldehyde. The formation of formaldehyde and formic acid as major products is consistent with a mechanism involving α-scission between carbons C1–C2 and C2–C3 of glycidic units, leading to the sequential formation of formaldehyde and its subsequent oxidation to formic acid.^52^

The total amount of hydrogen produced (262.5 μmol) corresponds to approximately 3.9 mmol of H_2_ per mmol of CLB converted, while the products quantified in the liquid phase represented approximately 1.4 mmol L^–1^ of carbon. Although the carbon balance was not fully closed under the present analytical framework, the detected products, particularly formic acid and formaldehyde, are consistent with the occurrence of oxidative cleavage reactions during CLB photoreforming. While the quantified products account for only part of the converted carbon, their distribution is consistent with a deep oxidative cleavage pathway, although additional analyses would be required to fully elucidate the reaction mechanism and establish a complete carbon balance.

It should also be noted that CLB conversion after 3 h reaction time was only 5.4%, indicating limited substrate utilization under the investigated conditions. Although the catalyst showed stable hydrogen production and good operational performance, improving CLB conversion remains a key challenge and should be a focus of future optimization studies.

## 4 Conclusions

In this study, TiO_2_@Pt­(0.5) was successfully immobilized on glass plates via spray coating and evaluated for the photocatalytic reforming of CLB. Under optimized conditions, the immobilized system achieved a H_2_ production rate of approximately 14 μmol cm^–2^ h^–1^. When compared under the same catalyst mass and reaction conditions, the immobilized configuration exhibited hydrogen production comparable to that obtained in the slurry system, while offering the operational advantage of simplified catalyst recovery and reuse. Hydrogen evolution was primarily dependent on photon energy and catalyst loading, with an optimal mass of 14.5 mg beyond which no further increase was observed, likely due to limitations in light penetration and mass transport. The cumulative hydrogen yield obtained after 3 h of reaction was similar within the investigated temperature range (40–80 °C); however, operation at 80 °C resulted in a distinct initial kinetic profile and a small loss of immobilized catalyst, indicating that elevated temperatures may affect the mechanical stability of the catalytic film. The catalyst maintained stable activity over 10 reuse cycles, with no measurable mass loss. Although no significant deactivation was observed during reuse experiments, Pt leaching was not directly quantified and was therefore assessed only indirectly through catalyst mass measurements and photocatalytic performance. Liquid-phase analysis identified formic acid and formaldehyde as the main byproducts. These products are consistent with oxidative cleavage reactions during CLB photoreforming; however, the incomplete carbon balance prevents definitive mechanistic conclusions. In addition, CLB conversion remained limited (5.4% after 3 h), indicating that substrate utilization is still a major challenge under the investigated experimental conditions. Therefore, the present results should be regarded as a promising proof-of-concept demonstrating the feasibility of an immobilized TiO_2_@Pt­(0.5) photocatalytic system for CLB photoreforming rather than as evidence of a mature or scalable technology. Future studies are required to improve substrate conversion, close the carbon balance, directly assess Pt leaching, and optimize the catalyst-film architecture, reactor design, and optimizing operating conditions. Such efforts will be essential to establish the practical potential of this immobilized photocatalytic approach and to advance the mechanistic understanding of the process.

## Supplementary Material





## Notes

Scanning transmission electron microscopy (STEM) images and EDX mapping of the catalyst, along with catalytic plates images and measurements of the immobilized film thickness; textural properties and emission spectrum of the catalyst; and influence of excitation wavelength and temperature on photocatalytic activity (PDF)
H_2_ production via CLB photoreforming (immobilized plate catalyst) (MP4)
