# Supplementary material for: Photocatalytic Hydrogen Production from Cellobiose Using TiO2@Pt(0.5) Immobilized Photocatalyst
Source: ACS Omega. 2026 Aug 9;11(33):49214–24. doi: 10.1021/acsomega.6c01812 (PMC13520490; doi:10.1021/acsomega.6c01812)
Supplement: Supplementary file 1 [file ao6c01812_si_001.pdf]

Supporting information

Photocatalytic Hydrogen Production from Cellobiose Using TiO_2@Pt(0.5) immobilized photocatalyst

Bruno C. B. Salgado[1,4]; Mayara M. R. Oliveira[1]; Sydney F Santos[2]; Jeremy W. J. Hamilton[3]; Kathryn Ralphs[4]; Peter K. J. Robertson[4*]*

[1] Federal Institute of Ceara. Av. Parque Central, 1315, Maracanau, Ceara, 61939-140, Brazil

[2] Federal University of ABC. Av. dos Estados, 5001, Bangu, Santo André, São Paulo, 09.280-560, Brazil,

[3] School of Engineering, Ulster University, 2-24 York Street, Belfast, BT15 1AP, United Kingdom

[4] School of Chemistry and Chemical Engineering, Queen's University Belfast, Stranmillis Road, Belfast, BT9 5AG, United Kingdom

*Corresponding Authors:

Bruno C. B. Salgado, e-mail: brunocesar@ifce.edu.br

Peter K. J. Robertson, e-mail: p.robertson@qub.ac.uk

Summary

Figure S1. HAADF-STEM (a,b) images and EDX mapping (c-f) of the TiO_2@Pt(0.5). 3

Figure S2. N_2 adsorption/desorption isotherms and pore size distribution curves (insert) of TiO_2 (black) and TiO_2@Pt(0.5) (blue). 4

Figure S3. High resolution XPS spectra for Ti 2p for TiO_2 (black) and TiO_2@Pt(0.5) (blue). 5

Figure S4. Emission spectra of TiO_2 P25 (black) and TiO_2@Pt(0.5) (blue)............................6

Figure S5. Effect of lamp wavelength on photocatalytic performance for H_2 production. Catalyst: TiO_2@Pt(0.5), [CLB] = 50 mM, immobilized catalyst mass = 19.4 mg, T = 40 °C. .7

Figure S6. Images of photocatalytic plates prepared by spray coating with different immobilized masses of TiO_2@Pt(0.5). 8

Figure S7. Film thickness obtained by SEM from the cross-section of the plate with 14.5 mg of TiO_2@Pt(0.5). 9

Figure S8. Effect of temperature on the photocatalytic performance for H_2 production. Catalyst: TiO_2@Pt(0.5), [CLB] = 50 mM, immobilized catalyst mass = 14.5 mg, lamp 370 nm. 10

Table S1. Textural characteristics of TiO_2 and TiO_2@Pt(0.5). 11

Table S2. Atomic composition of TiO_2@Pt(0.5) obtained by XPS. 12

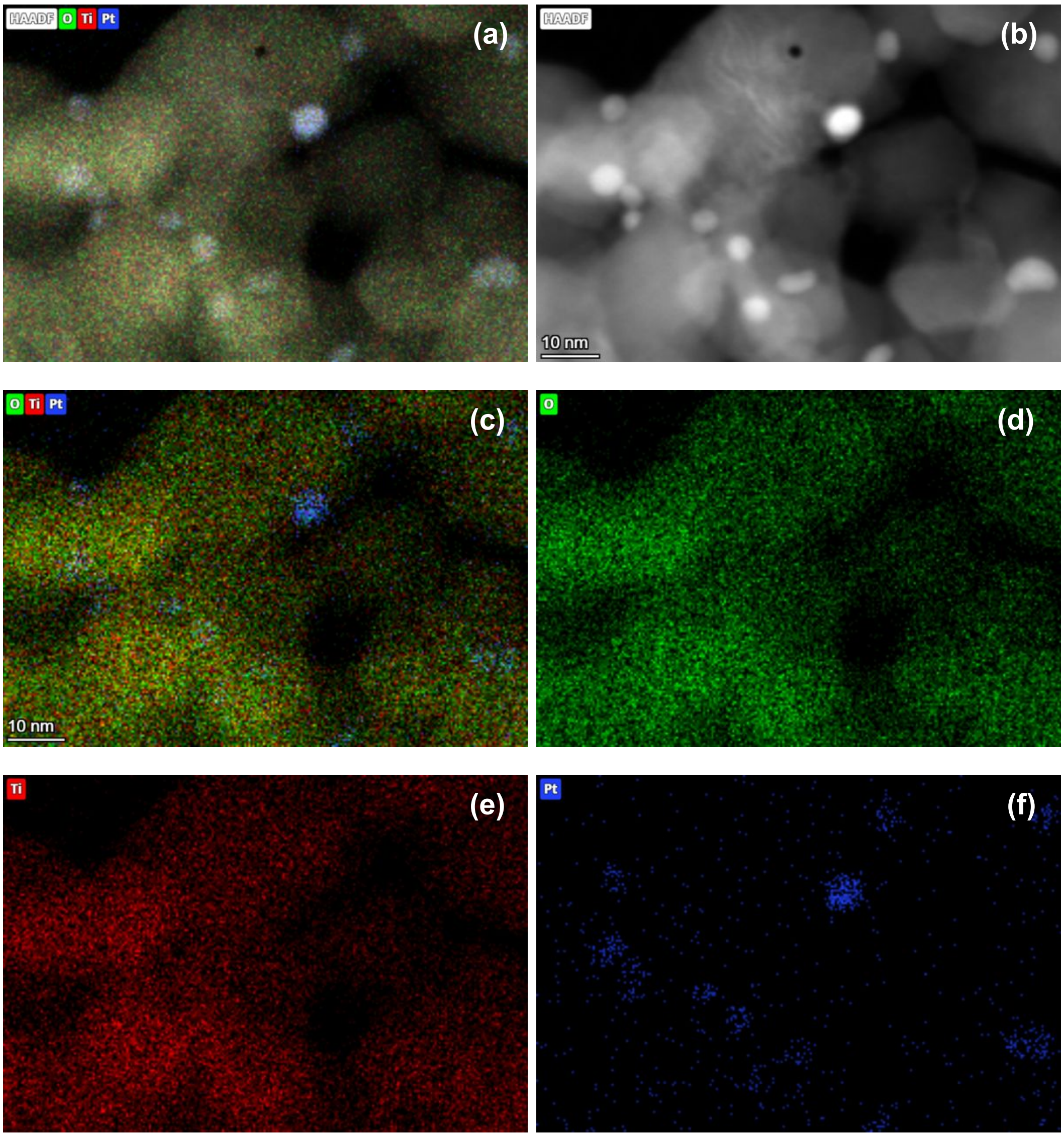

Figure S1. HAADF-STEM (a,b) images and EDX mapping (c-f) of the TiO_2@Pt(0.5).

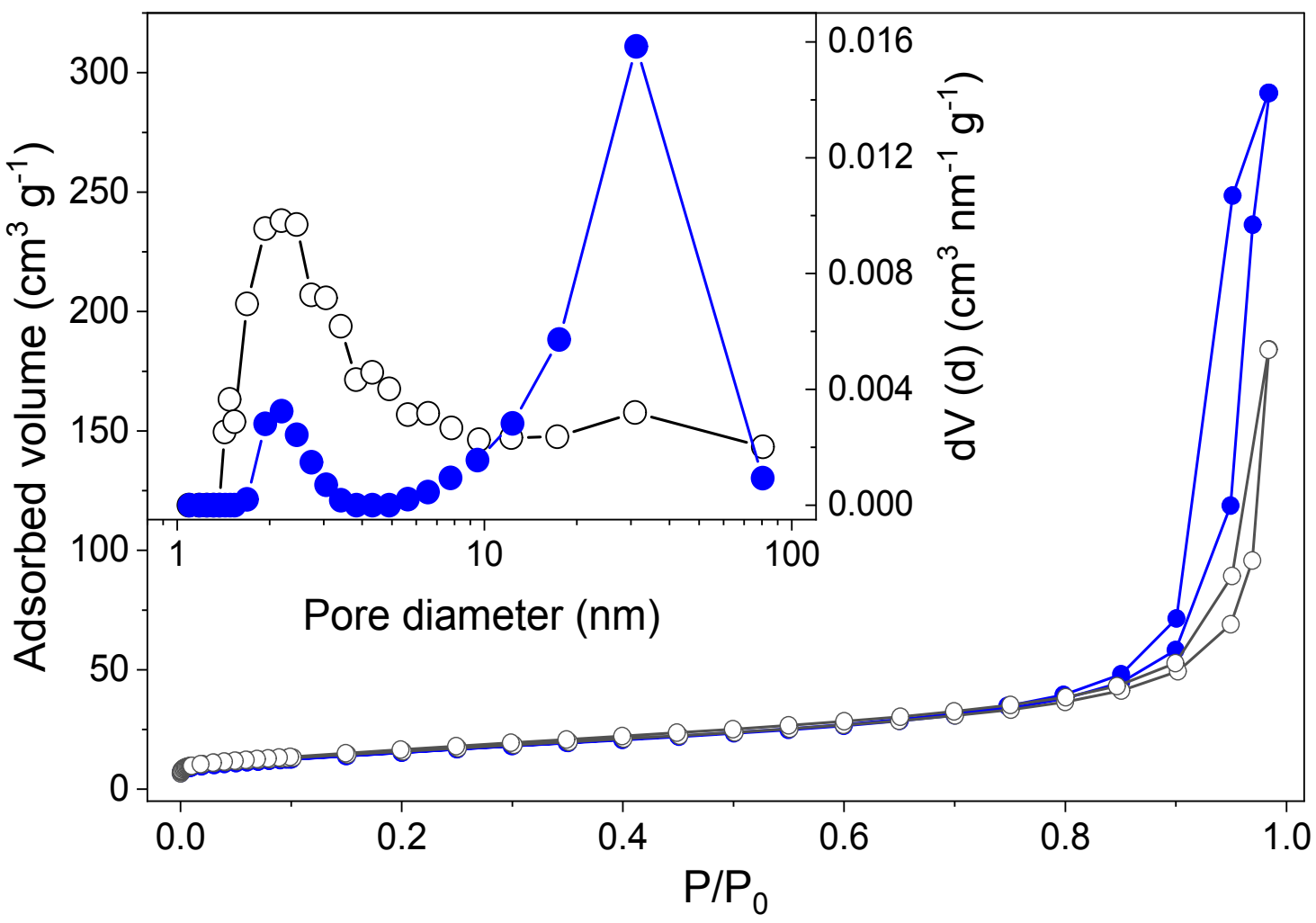

Figure S2. N_2 adsorption/desorption isotherms and pore size distribution curves (insert) of TiO_2 (black) and TiO_2@Pt(0.5) (blue).

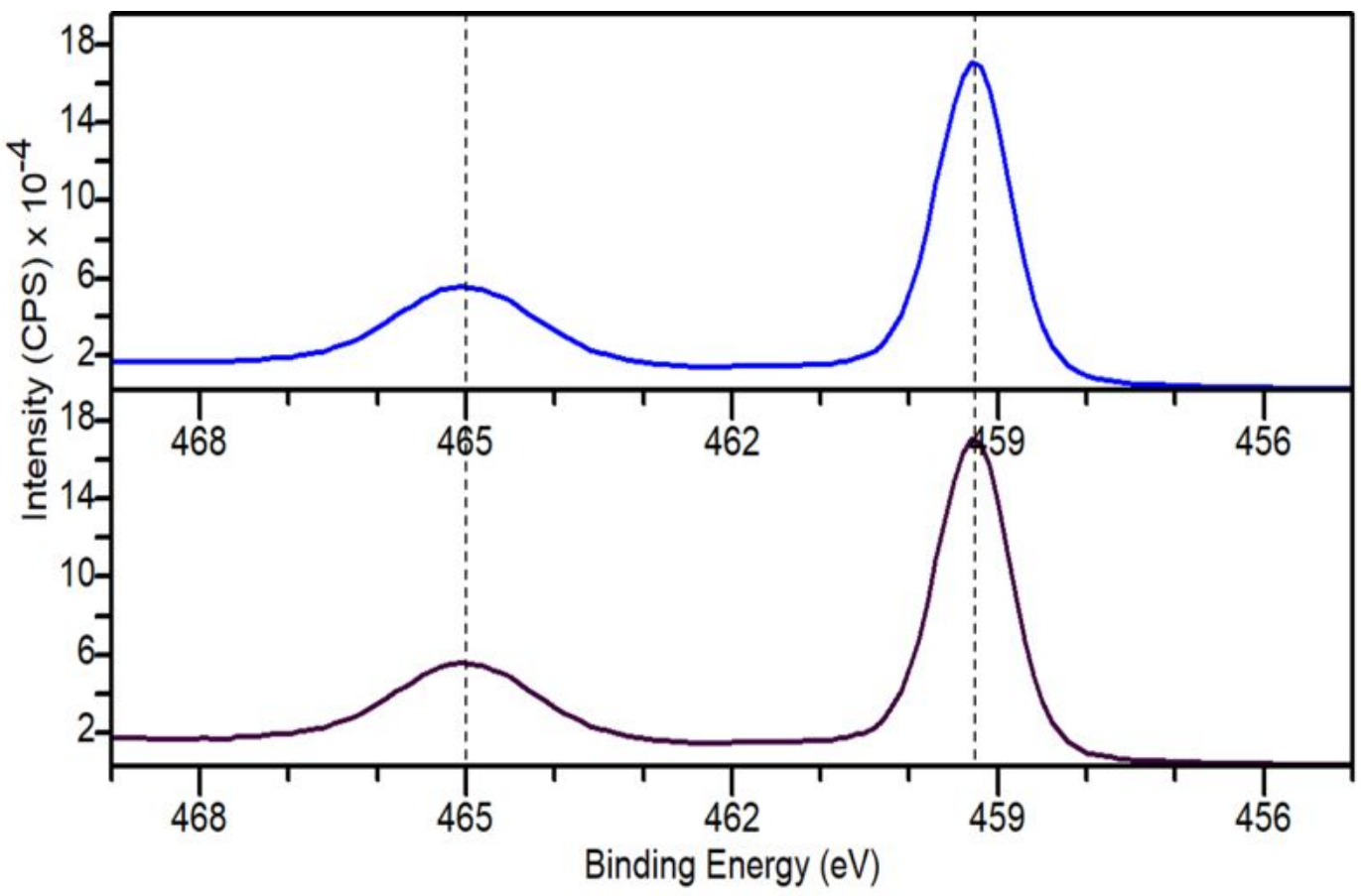

Figure S3. High resolution XPS spectra for Ti 2p for TiO_2 (black) and TiO_2@Pt(0.5) (blue).

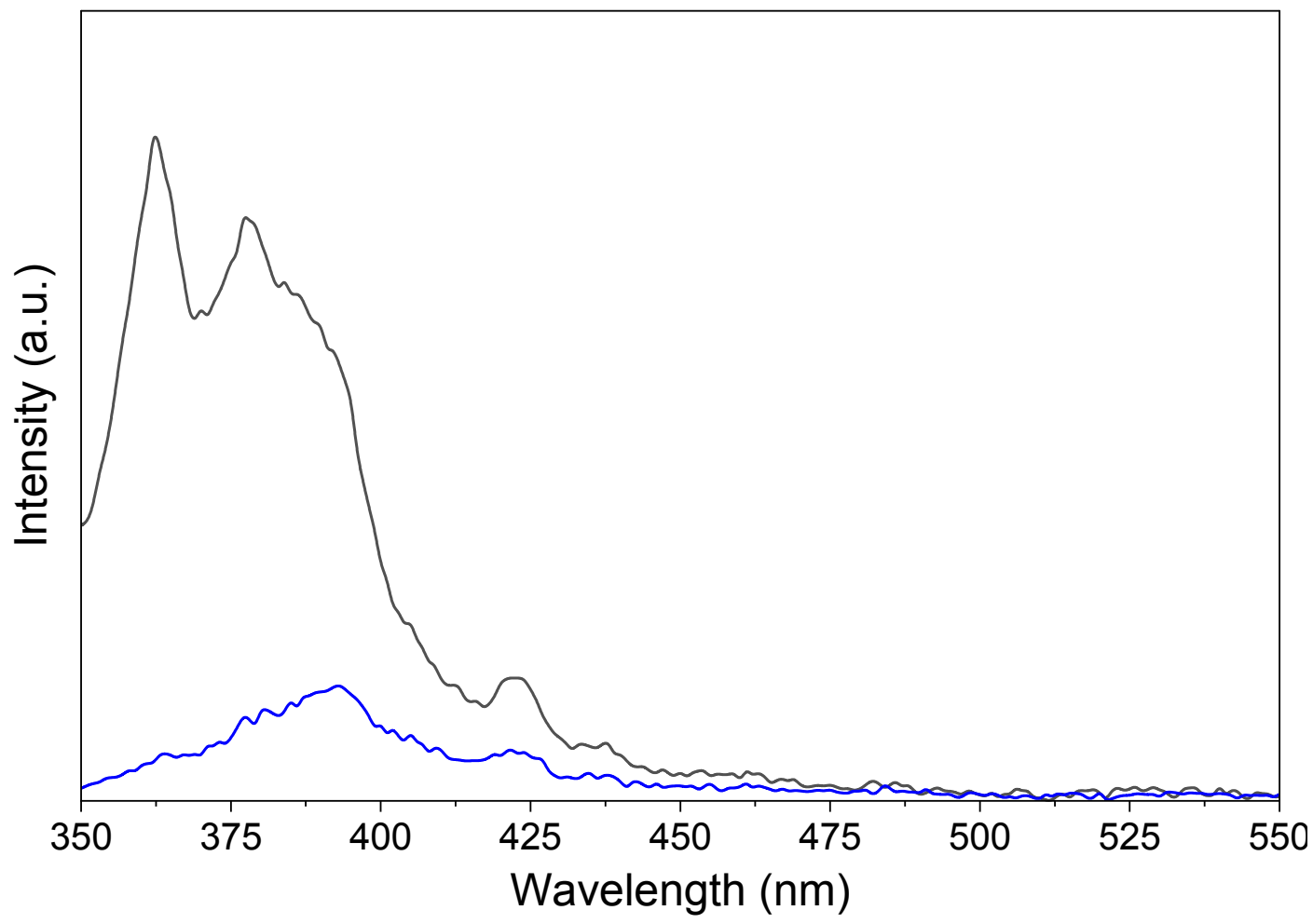

Figure S4. Emission spectra of TiO_2 P25 (black) and TiO_2@Pt(0.5) (blue).

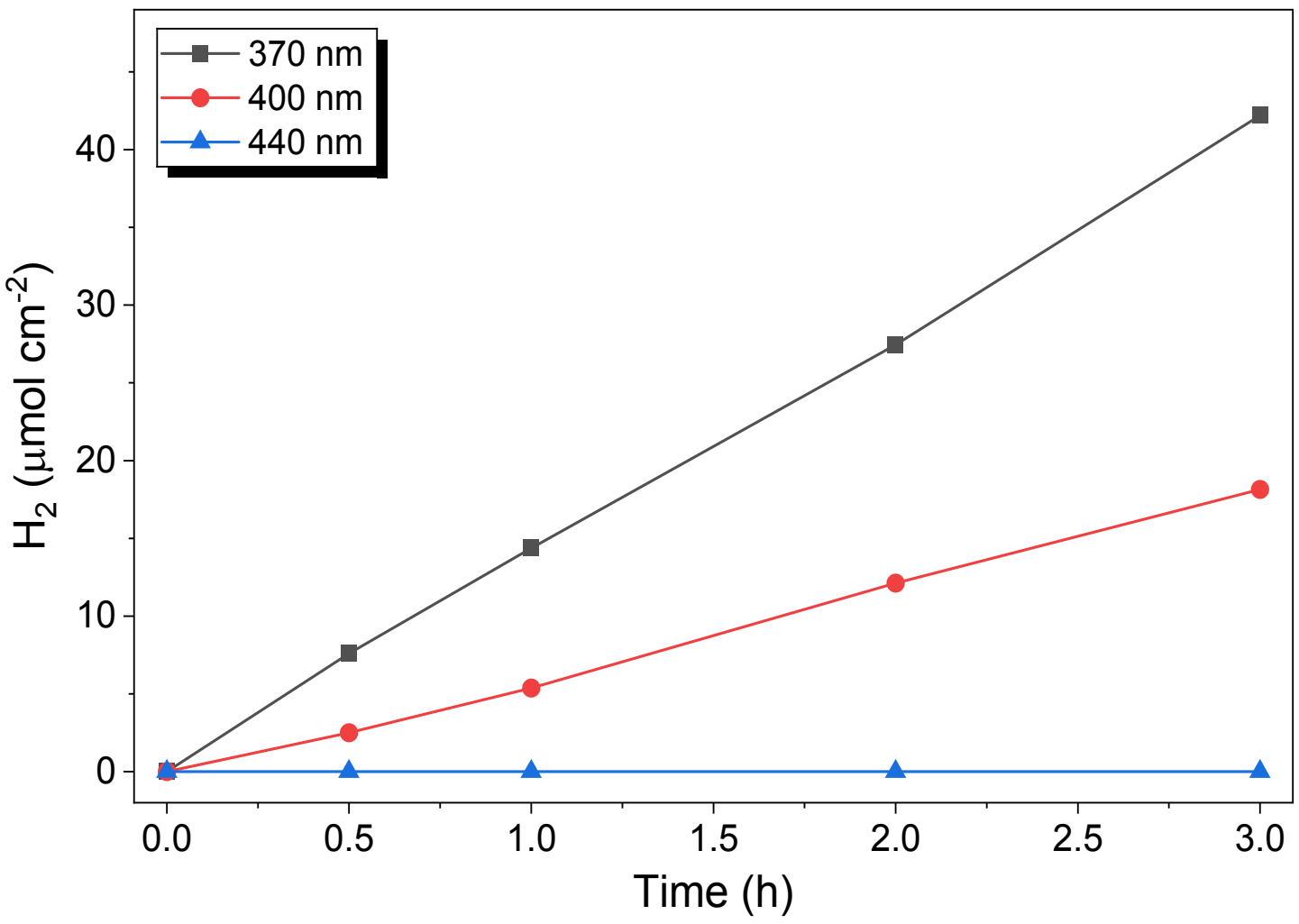

Figure S5. Effect of lamp wavelength on photocatalytic performance for H_2 production. Catalyst: TiO_2@Pt(0.5), [CLB] = 50 mM, immobilized catalyst mass = 19.4 mg, T = 40 °C.

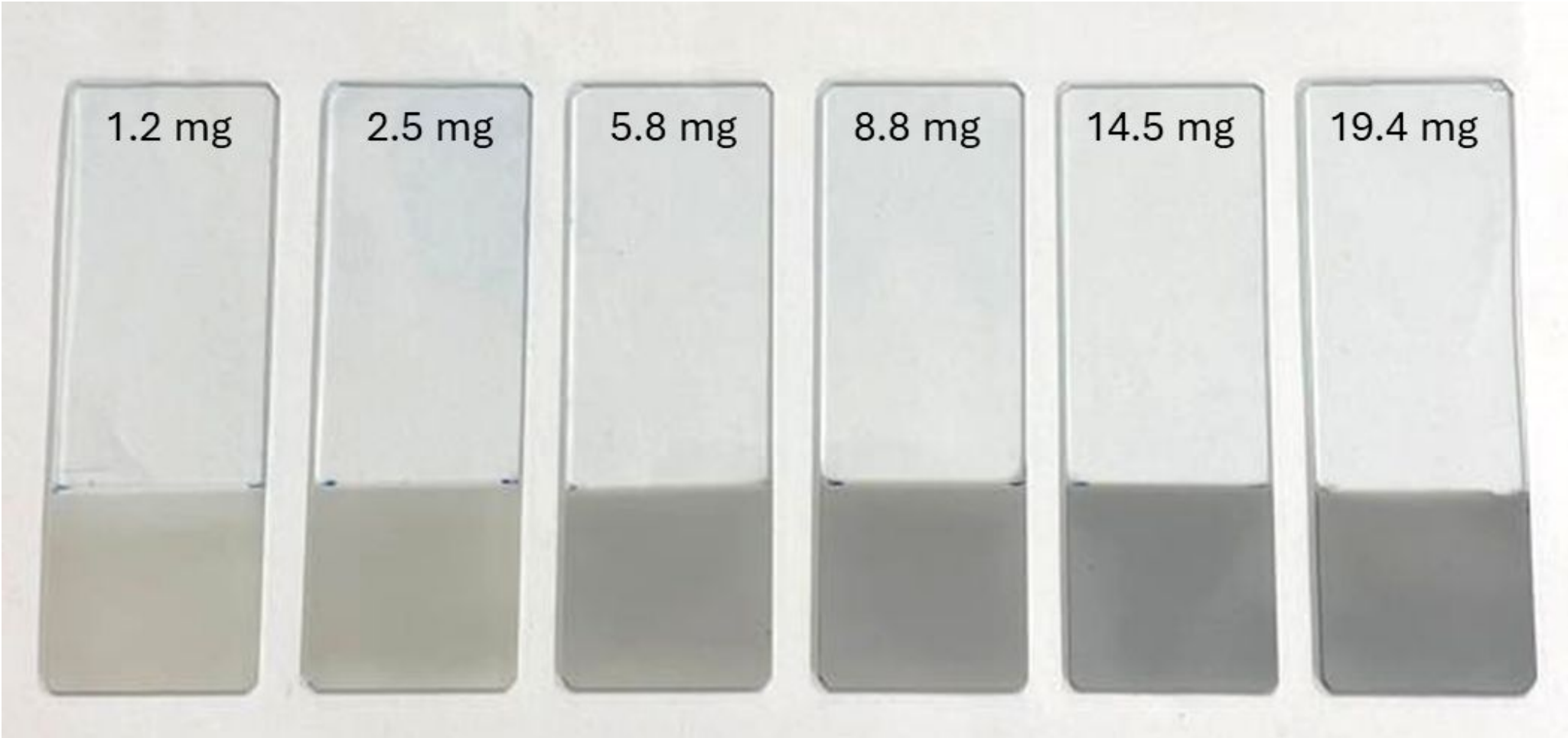

Figure S6. Images of photocatalytic plates prepared by spray coating with different immobilized masses of TiO_2@Pt(0.5).

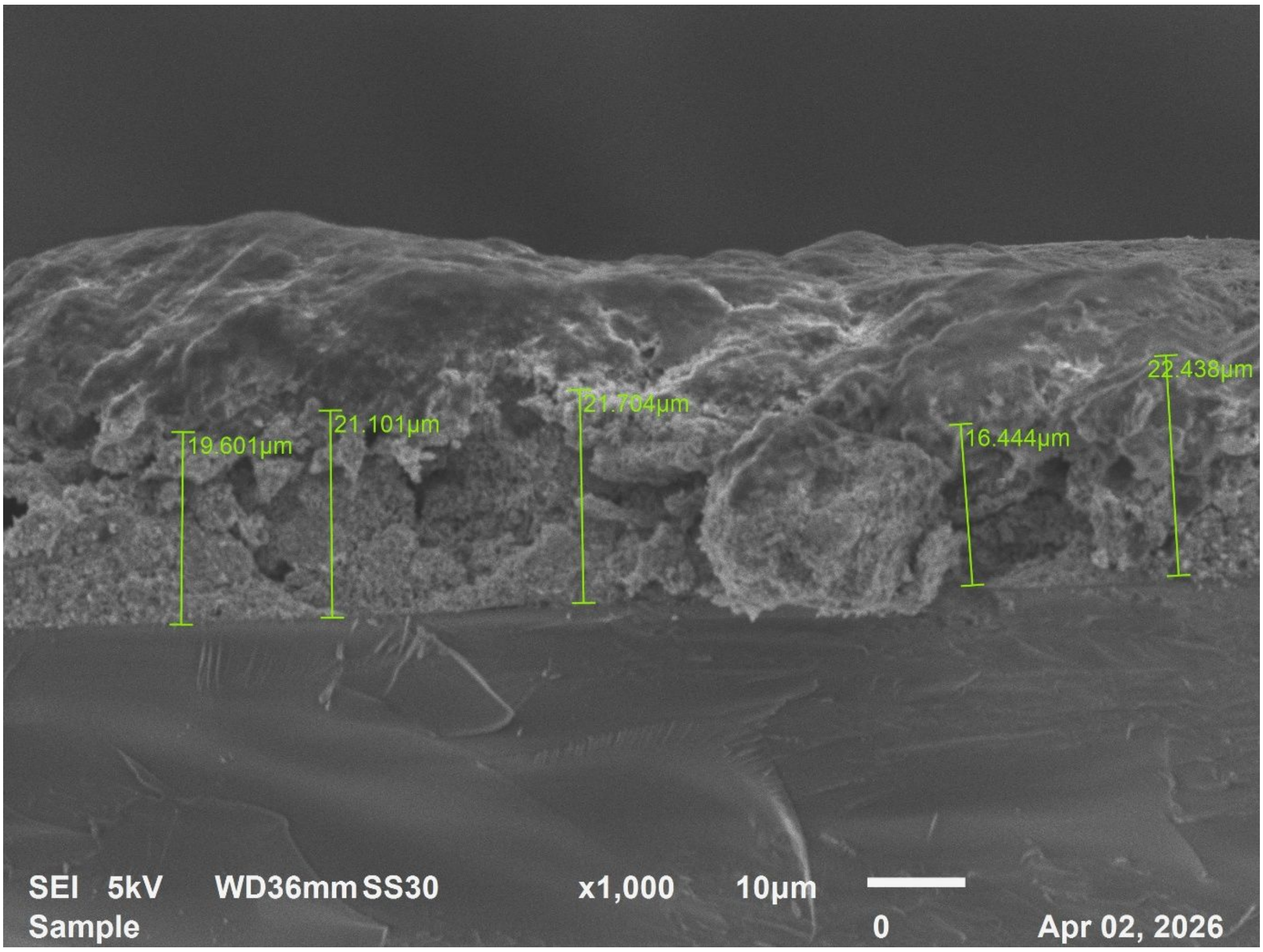

Figure S7. Film thickness obtained by SEM from the cross-section of the plate with 14.5 mg of TiO_2@Pt(0.5).

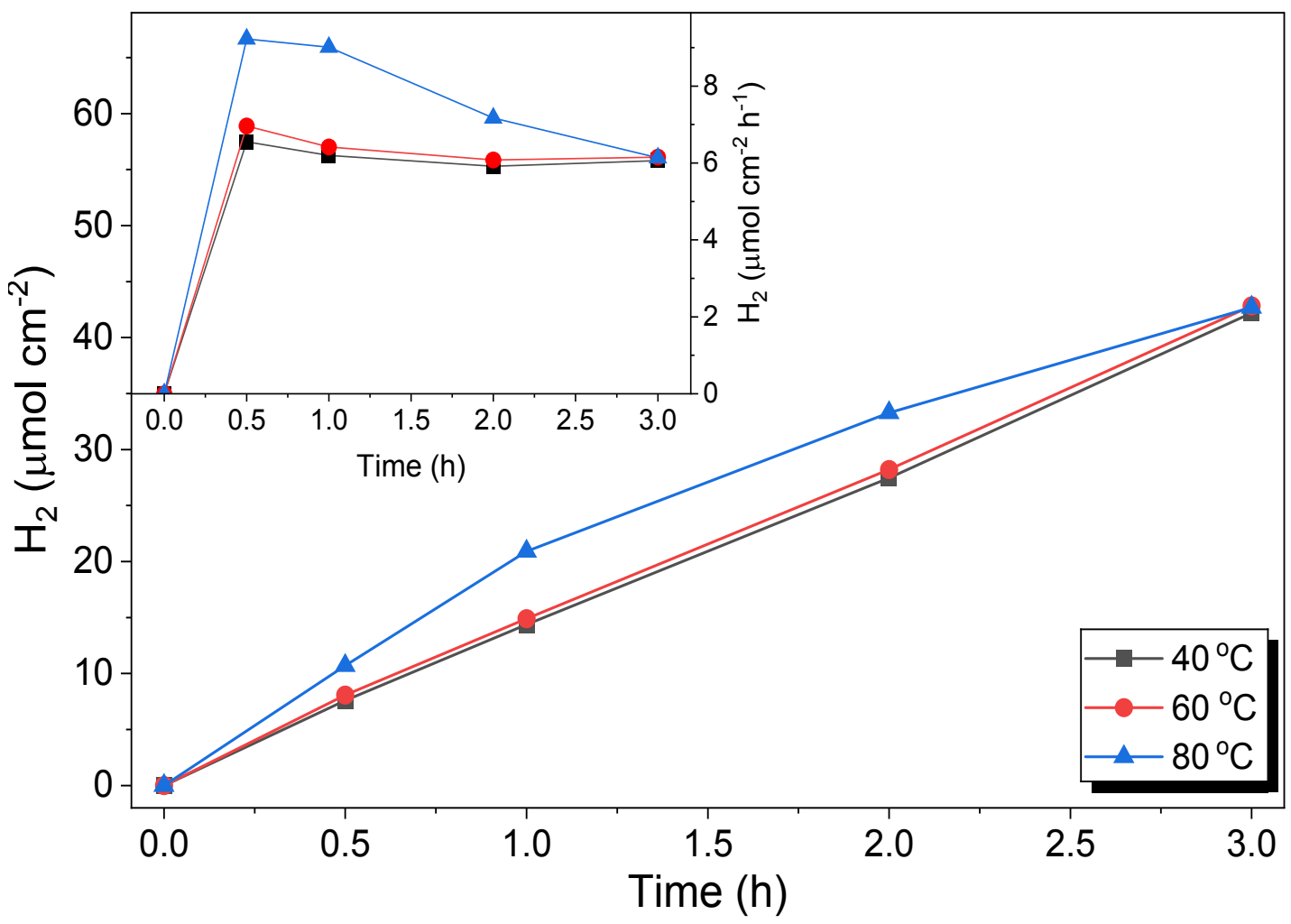

Figure S8. Effect of temperature on the photocatalytic performance for H_2 production. Catalyst: TiO_2@Pt(0.5), [CLB] = 50 mM, immobilized catalyst mass = 14.5 mg, lamp 370 nm.

Table S1. Textural characteristics of TiO_2 and TiO_2@Pt(0.5).

Catalyst	Specific surface area (m^2 g^{-1})	Pore diameter (nm)	Pore volume (cm^3 g^{-1})
TiO_2 P25	64.97	2.186	0.286
TiO_2@Pt(0.5)	65.18	31.216	0.456

Table S2. Atomic composition of TiO_2@Pt(0.5) obtained by XPS.

Region	Binding energy (eV)	Atomic percentage (%)
Ti 2p	458.81	27.22
O 1s	530.03	67.85
C 1s	284.89	4.71
Pt 4f	74.19	0.23
